# Drivers of piscivory in a globally distributed aquatic predator (brown trout): a meta-analysis

**DOI:** 10.1038/s41598-020-68207-8

**Published:** 2020-07-09

**Authors:** Javier Sánchez-Hernández

**Affiliations:** Área de Biodiversidad y Conservación, Departamento de Biología y Geología, Física y Química Inorgánica, Universidad Rey Juan Carlos, Móstoles, Madrid Spain

**Keywords:** Freshwater ecology, Ichthyology

## Abstract

There is growing interest in the delineation of feeding patterns in animals, but little is known about the interaction of multiple explanatory factors across broad geographical scales. The goal of this study was to identify the factors that together determine population-level patterns in piscivory in a globally distributed aquatic predator, the brown trout (*Salmo trutta*). A meta-analysis of peer-reviewed studies revealed that the prevalence (frequency of occurrence, %) of piscivory increases from riverine to marine ecosystems, with fish community type and the size-structure (ontogeny) of brown trout populations being the key drivers. Thus, piscivory was related to ecosystem-specific differences in predator body size (increasing in populations with large individuals) and fish community configurations (increasing with fish species richness). Fish species richness imposes important limitations on (i.e. in low diversity scenarios) or facilitate (i.e. in high diversity scenarios) piscivory in brown trout populations, with a low prevalence expected in low-diversity fish communities. In fresh water, piscivory is higher in lentic than lotic ecosystems and, in the former, increases with latitude. Competition in multi-species systems is expected to be higher than in simpler systems because the size-structure and species composition of fish assemblages, explaining cross-ecosystem differences in piscivory.

## Introduction

Understanding piscivory has the potential to provide fundamental insights into important inter-dependent linkages among individual, population and community levels. Piscivorous fish species can have a significant influence on the abiotic (suspended solids and total phosphorus concentrations) and biotic (chlorophyll-*a* and community structure) components of aquatic ecosystems through predator–prey interactions and top-down mechanisms^1–3^. Piscivory has a direct impact on fish communities (e.g. through changes in fish abundance, biomass, or population or community structure), which can lead to reductions in suspended solids, phytoplankton biomass and eutrophication^2^. In addition, piscivory plays an important role at the individual level, as fish that transition to piscivory early may benefit from higher somatic growth, earlier maturation and higher fitness^4^.

Many factors, including ontogeny^5^, temperature^6,7^, competitive scenarios^5,8^, availability of vulnerable prey sizes^5,9^ and community structure or fish species richness^5,8^, have been hypothesised to affect the prevalence of piscivory in fish populations. Because many fish species are gape-limited predators, and mouth gape scales with body size, much attention has been paid to the size-related timing of the switch to piscivory^4^. The onset of piscivory seems to be linked to high water temperatures in early development^10^, but also seems to be dependent on resource competition with other fish species and the presence of small prey fish^5^. Thus, the relationship between predator and prey body size is particularly important in understanding the prevalence of piscivory in fish populations as the presence of suitably sized fish prey promotes piscivory in top fish predators^5,9,11,12^. Intraguild predation (size-structured mixed competition–predation interactions) can also be an important factor where transitions to piscivory can be displayed because fish species interact as predator and prey^5,13,14^.

The proportion of piscivorous species is greater in marine than in freshwater systems^15^. Prevalence of piscivory within species can also vary geographically, being highest at high latitudes in both aquatic mammals^16^ and fishes^6,7,17^ (but only demonstrated through cannibalism in fish). It is thought that a lower availability of alternative prey (other than fish) favours piscivory at higher latitudes compared with diverse diets at lower latitudes in both riverine and lacustrine systems^12,13^. Thus, latitudinal variation in the prevalence of piscivory by aquatic predators might be connected to one of the most outstanding ecological patterns in nature, the latitudinal gradient of species diversity, with more diverse ecosystems towards the equator^18^. Fish consumption is inversely correlated with altitude in freshwater mammals as a consequence of decreases in fish availability with altitude^19^. However, most studies of piscivory have focussed on simple approaches exploring variation among local systems and during ontogeny^4,11,20^, and lack broad biogeographic perspectives, inter-ecosystem comparisons and multifactor approaches to explore the combined effects of the multiple factors that can influence piscivory^5,8^. Herein, I look beyond simple cause-and-effect associations and focus on an holistic understanding of variation in piscivory among fish populations, based on the integration of multiple factors, through the central question: what ecological factor (fish community type, fish prey system, fish population size-structure, temperature, altitude and latitude) or combination of factors determines the prevalence of piscivory at the population level?

I address this question by using a multifactor approach, with brown trout (*Salmo trutta* Linnaeus, 1758) as model organism, to identify the variables that are the most influential drivers of piscivory. The model species is indigenous to Europe, North Africa and western Asia, where it inhabits riverine, lacustrine and marine ecosystems, but has been introduced to, and become invasive in, many countries outside its native range^21^. This wide geographic distribution and use of multiple ecosystem types facilitates the use of brown trout as a model organism to establish whether patterns in the trophic ecology (here piscivory) of fishes can be extended to an ecosystem dimension. Additionally, brown trout has an outstanding socio-economic importance, both in commercial and sport fisheries^22^. As a consequence, the species has caught the attention of many researchers^21^, which has led to a good understanding of its trophic ecology^12,23,24^ and an extensive literature suitable for systematic reviews and meta-analyses. The species is also a top predator and undergoes ontogenetic dietary shifts from aquatic invertebrates to fish prey, with the transition to piscivory in lacustrine food webs promoted by the presence of suitably sized fish prey^5,11,12^. This paper explores population-level differences in the prevalence of piscivory (hereafter piscivory) among three contrasting ecosystem types (riverine, lacustrine and marine), aiming to disentangle the magnitude and direction of any ecosystem-level dissimilarities in piscivory in the model organism. The hypotheses were thus that (i) ecosystem type plays a key role in determining the population-level of piscivory and (ii) for a given ecosystem type, the population-level of piscivory will increase with increasing fish species richness, brown trout length and temperature-related geographical (i.e. increasing latitude and decreasing altitude) variables. I anticipated that the population-level of piscivory would increase from freshwater to marine ecosystems, and that piscivory was expected to be highest in diverse fish communities containing large brown trout, and lowest in allopatric populations containing only small brown trout. This study also aims to corroborate whether feeding patterns (here piscivory) can be linked to latitudinal and elevational gradients in species richness (i.e. species richness decreasing with increasing latitude and altitude^18,25^).

## Results

**Figure 1:**
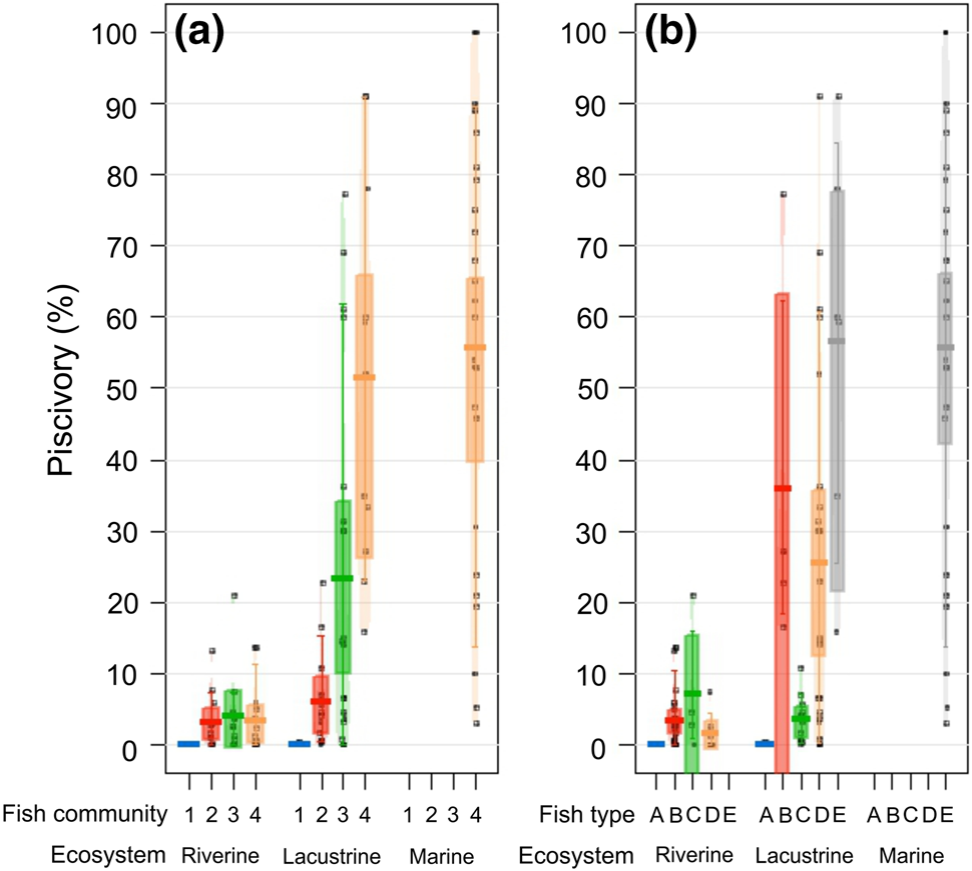
Violin plots (box plots with the probability density) showing the prevalence of piscivory (%) of brown trout according to ecosystem type nested with (**a**) fish community type (1 = one-species system, 2 = two-species system, 3 = three-species system, 4 = multi-species system) and (**b**) fish prey type (A = cannibalism, B = fish prey, C = potential fish competitor, D = fish prey and potential competitors, and E = fish prey, competitors and predators). The boxplot within each violin plot indicates the median and the interquartile range with the 95% confidence interval for the median.

Two models with substantial support were selected according to AICc, of which both included three predictor variables (body size, latitude and elevation) and two interaction terms (body size − fish prey type and latitude − ecosystem type × fish community type) (Table 1 and Supplementary Appendix S1). According to the residuals, the best models captured the patterns in the data reasonably well and seemed to be reliable, despite a small amount of spatial autocorrelation in the residuals (Supplementary Appendix S2). The population-level of piscivory increased with increasing number of sympatric fish species, which was clearly observed in lacustrine ecosystems (Fig. 1a) and supported by within-ecosystem pairwise comparisons (Supplementary Appendix S3). One-species systems had lower piscivory compared to the other fish community types (Supplementary Appendix S3). In lacustrine ecosystems, piscivory was higher in fish prey communities than in communities composed of only potential fish competitors (Mann–Whitney–Wilcoxon, *W* = 44, *P* = 0.001, Fig. 1b). Regarding between-ecosystem pairwise comparisons, statistically significant differences were found between three- (*W* = 52.5, *P* = 0.018) and multi-species (*W* = 10, *P* < 0.001) riverine and lacustrine ecosystems, and also between multi-species riverine and marine ecosystems (*W* = 10, *P* < 0.001). Piscivory was higher in lacustrine than in riverine ecosystems in fish prey systems (*W* = 5, *P* = 0.002) and two-prey (prey and potential competitors) systems (*W* = 23, *P* = 0.005) (Fig. 1b).

**Table 1:** Significance table of the best generalised additive mixed model simulations (GAMMs) for prevalence of piscivory (%) according to AICc values (see model selection tables including the 10 best model simulations is shown in Supplementary Appendix S1).

	Full model	Sensitivity analyses	
		Piscivory (only mean body size)	Piscivory (only multispecies systems)
**Parametric coefficients**			
Intercept	45.380*	− 9.048	− 17.506*
Latitude	− 1.172**	−	−
Body size	0.061***	−	0.077***
**Smooth terms (Edf)**			
Body size − fish prey type	+**	−	−
Latitude − ecosystem type × fish community type	+***	+***	+***
**Model statistics**			
R^2^	0.70	0.56	0.71

*Edf* estimated degree of freedom for smooth terms are shown.

***P < 0.001, **P < 0.01 and *P < 0.05.

**Figure 2:**
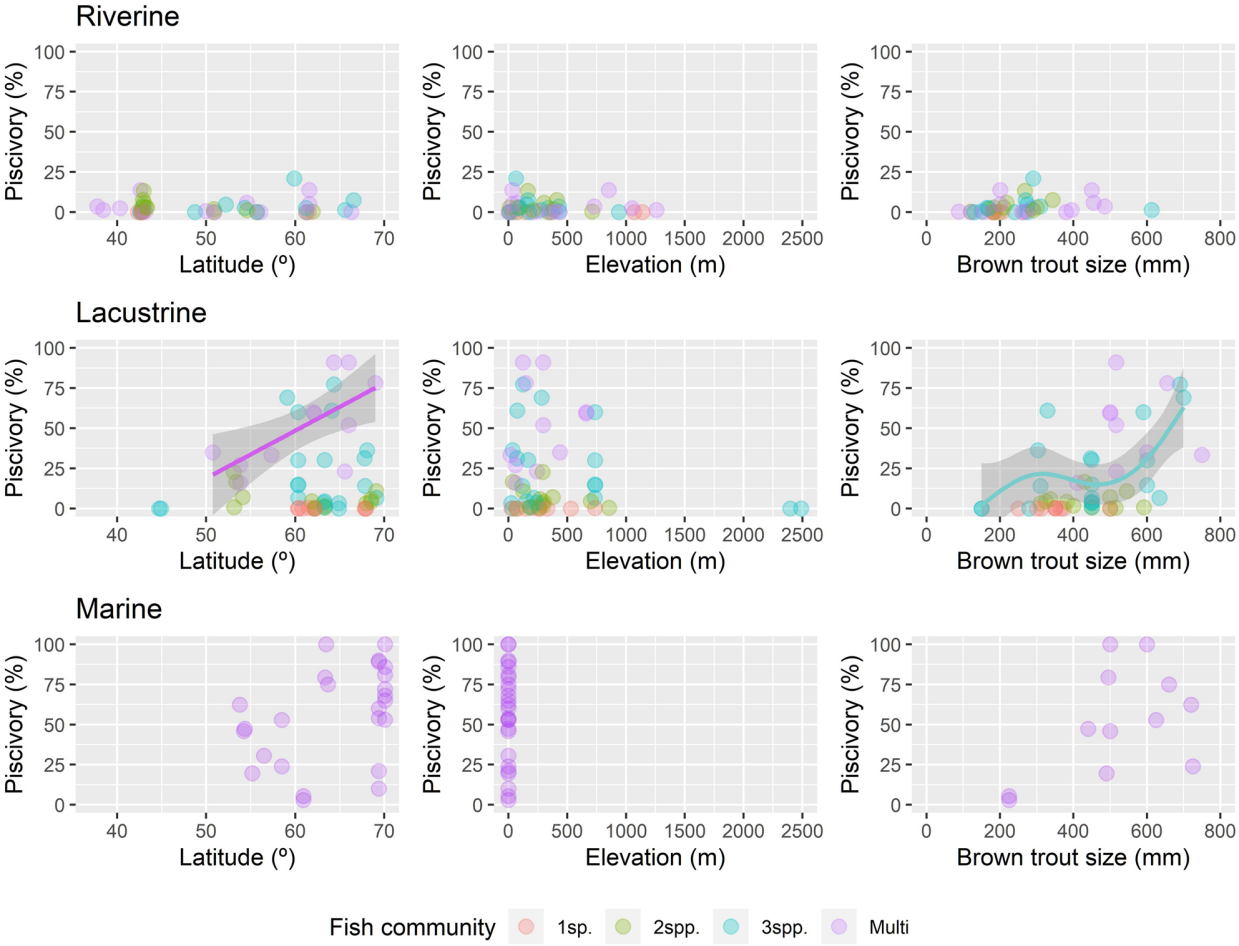
Piscivory along covariates (latitude, elevation and maximum body size of brown trout). Data are displayed for type of ecosystem (riverine, lacustrine and marine) and type of fish community (one-species system, two-species system, three-species system, and multi-species system). Fitted lines (method = “gam”) with 95% confidence intervals are only shown for statistically significant relationships according to Supplementary Appendix S4.

Sensitivity analyses (only including multi-species systems and mean brown trout length) corroborated the importance of ecosystem type, fish prey type, brown trout body size and latitude as drivers of piscivory (Table 1). However, the best models recognised only three-way interactions (latitude − ecosystem type × fish community type), which suggests that the effect of latitude on piscivory was different at different levels of ecosystem type (Fig. 2) and fish community type (Supplementary Fig. S4B).

Despite inclusion as a predictor in the models with substantial support (Supplementary Appendix S1), there were no clear patterns in the response of piscivory to elevation (Fig. 2). The relationship between piscivory and latitude varied according to ecosystem and fish community type, with piscivory increasing with increasing latitude only in multi-species, prey fish and three-prey systems of lacustrine ecosystems (Fig. 2 and Supplementary Appendix S4). Piscivory increased with increasing brown trout body size in lacustrine ecosystems (Supplementary Appendix S4) and three-species systems of lacustrine ecosystems (Fig. 2).

## Discussion

This study provides evidence that the population-level of piscivory increases from lotic to lentic ecosystems. Fish community configuration and brown trout population structure are the most influential drivers of variation in the prevalence of piscivory across ecosystem types. Models also recognised the influence of temperature-related geographical (here latitude and elevation) variables on piscivory. However, the relationship between piscivory and latitude varied according to ecosystem and fish community type, as latitudinal variation in piscivory was only evident in lacustrine populations (for which piscivory increased with latitude). The influence of elevation was less important than that of latitude.

The hypothesis that piscivory increases from freshwater to marine ecosystems was only partially accepted because there were no clear differences in piscivory between lacustrine and marine populations; thus, the hypothesis was only supported for riverine populations (piscivory was lower in riverine than in lacustrine and marine ecosystems). Early work predicted that the proportion of piscivorous species is greater in marine than in freshwater systems, most likely because of an increasing gradient in fish species richness from fluvial headwaters to the sea^15^. However, the authors identified that the overall prevalence of piscivory at the assemblage level was highest in brackish coastal lagoons, and similar between riverine and marine systems (see Table 4 in Winemiller and Leslie^15^). The present study suggests otherwise, demonstrating clear differences in piscivory at the population level between riverine and marine systems, but only in multi-species systems as marine ecosystems were only composed by this fish community configuration as a consequence of the difficulties of finding local fish communities composed by few species in the study area^26,27^. It should be noted that the feeding patterns of fish in brackish systems are dissimilar to those observed in freshwater systems as consequence of differences in the size, abundance and diversity of both invertebrate and fish prey^9,28^. Thus, it is possible that the different biological level of organisation (population in the current study and assemblage in Winemiller and Leslie^15^), but also differences in productivity and species composition related to geographical location (tropical areas of Costa Rica) and a disparity in sampling effort among ecosystem types (only 733 ocean individuals compared with 5,485 lagoon individuals), explain the different outcomes found by Winemiller and Leslie^15^ and the current study. Analogous studies focussed on fish cannibalism have concluded that cannibalism can be assumed to be more frequent in freshwater than in marine systems because cannibalism is, in general, more frequent in communities with lower fish species richness and in small and more closed to dispersal systems (freshwater fish assemblages seem to be dispersal limited compared to marine fish assemblages)^7^. However, other studies have found that cannibalism can be similar, in terms of number of literature reports of cannibalism, between marine and freshwater fishes^29^. These are seemingly contradictory standpoints, but the apparent contradictions can be resolved if taxonomy (i.e. taxonomic relatedness of species) and prey availability is taken into account because: (i) dietary habits may have significant associations with the phylogenetic–taxonomic relatedness of species^30–32^, (ii) piscivory seems to be a common feature when suitably sized fish prey are available^5,9,10^ and (iii) cannibalism is more likely to occur in situations with no suitably sized fish prey of other species^9,10^. It is possible that data biases caused by uneven fish community representations across the different ecosystem types may hinder our ability to identify broader gradients in piscivory across ecosystem types. However, this study provides evidence that piscivory increases from lotic to lentic ecosystems when comparing similar fish community configurations (here multi-species systems).

The current study shows how fish community configuration can trigger piscivory regardless of ecosystem type. For example, piscivory in lacustrine populations was always high in fish communities including fish prey (but remarkably low in allopatry and fish competitor systems) and increased with an increasing number of sympatric fish species. In contrast, piscivory in riverine populations showed no clear differences between fish prey systems, fish competitor systems or two-prey systems. It should be kept in mind that potential fish competitors (e.g. *Salvelinus* spp.) can also be both prey and predators for brown trout depending on size^5,33,34^, but the prevalence of piscivory of brown trout is usually very low in sympatry with *Salvelinus* spp.^5,34^. Thus, piscivory in fish competitor systems can be understood as consequence of intraguild predation as brown trout and many potential fish competitors rely on the same preferred prey resources (macrozoobenthos), and thus brown trout feed on potential fish competitors^5,13,14^. It is possible that the presence of fish prey has a greater influence on prevalence of piscivory in lacustrine populations compared to riverine populations, likely because lakes commonly support lower benthic macroinvertebrate production compared to rivers and streams^35^. The prevalence of cannibalism in the current study was very low, which is congruent with previous findings in riverine and lacustrine populations^36–38^. It is reasonable to posit that piscivory is higher in systems with several potential competitors and/or predators (highly competitive interactions) compared to systems where the model organism is the only piscivorous species. Competition in multi-species systems is expected to be higher than in simpler systems because the size-structure and species composition of prey fish assemblages have a direct effect on piscivory^5,39^. The evidence supports the conclusion that fish species richness imposes important limitations on (i.e. in low diversity scenarios) or might facilitate (i.e. in high diversity scenarios) piscivory in fish populations, with a low prevalence expected in low-diversity fish communities.

However, the understanding of piscivory should not be limited to fish species richness per se, but also contemplate small-bodied species or life stages (i.e. juveniles of large species)^5,9^. Thus, body size and ontogenetic mechanisms may also be major determinants of piscivory^4,5^. The current study shows that patterns in the trophic ecology of fish (here the prevalence of piscivory) can be related to ecosystem-specific differences in predator body size and fish community configurations (e.g. prey, competitor or predator), as indicated using a multifactor approach (Table 1). The observed differences in the prevalence of piscivory between lentic (both lacustrine and marine) and riverine ecosystems may be a result of larger individuals in lentic compared to riverine brown trout populations, and more similar size-structured brown trout populations between lacustrine and marine ecosystems could partially explain the similarity in piscivory in those ecosystems. However, the relationship between body size and piscivory is a clear example showing that processes scaling with body size may be driving a switch to piscivory (size-related dietary shifts) or, on the other hand, piscivory may be a consequence of the predator’s size-structure populations (populations composed by large individuals consume more fish prey); as most studies indicate that increases in growth rates can be caused by switches from invertebrates to fish^4^. Body size determines a suite of morphological traits (e.g. mouth gape and gill raker size or density) that can affect prey-handling characteristics during the lifetime of fish and which can impose important limitations on the timing and prevalence of piscivory in fish populations^4^. It is possible that predator body size is a key driver of piscivory in the early development of some species, but a consequence for adult individuals. In addition to predator body size, piscivory also depends on prey fish size distributions in terms of the availability of small-bodied species and juveniles of large species^5,9^. Indeed, predator–prey interactions are highly size-dependent^4,9^, but the lack of information about fish prey size in data sources prevented the exploration of predator:prey size ratios in the current study. However, it is important to note that brown trout become predominantly piscivorous at a minimum body length of 140–300 mm^5^, but it has been observed in individuals of smaller sizes (85 mm) in riverine and high-competition systems^40^. Despite cross-ecosystem differences in body size, the brown trout populations considered in this study can potentially feed on other fish and thus piscivory seems to be dependent upon cross-ecosystem differences in fish community configurations.

Temperature-related geographical (i.e. latitude and elevation) effects may also be major determinants of piscivory^7,17^. The prevalence of piscivory was associated with temperature-related geographical variables, but latitude emerged as a better predictor than elevation for uncovering geographical variations in the prevalence of piscivory in brown trout populations. Indeed, the prevalence of piscivory changed greatly according to elevation, unlike in aquatic mammals for which fish consumption can be negatively correlated with elevation^19^. Despite several studies supporting the view that piscivory and cannibalism increase with increasing latitude^6,7,16,17^, the current study suggests that latitude might not be a reliable predictor of the prevalence of piscivory in fish because it was masked by ecosystem and fish community type. This is possible because fish community configurations and the size structure of predator (and prey) populations differ along broad geographical ranges in line with latitudinal gradients in species richness^18^ and Bergmann’s rule^41^. Bergmann’s rule predicts that the maximum and age-specific body size of endothermic animals increases with increasing latitude, but the principle is controversial in ectotherms^42,43^. In this regard, the age-specific body size of stream-dwelling brown trout populations decreases with latitude^44^, whereas adult size in anadromous populations seems to be similar along latitudinal gradients^45^. On the basis that brown trout body size may change along latitude gradients, concomitant changes in the prevalence of piscivory should be expected. However, this study shows that despite the size-structure of brown trout populations being similar along latitudinal gradients (see Supplementary Appendix S5), the prevalence of piscivory increased with increasing latitude in lacustrine populations. This emphasises that latitude-dependent factors other than body size, such as fish community configurations, may display a key role in shaping geographic variation in piscivory. It is thought that piscivory in freshwater taxa is higher when the availability of alternative prey (other than fish) is low or in low-diversity ecosystems, such as at high latitudes^16,17^. This notion accepts the view that piscivory in fresh water is commonly promoted by low food availability and thus competitive scenarios. The current study identified consistent latitudinal gradients in multi-species systems of lacustrine populations, but not in riverine ecosystems, which underscores that piscivory in lake- and stream-dwelling populations may not respond similarly to latitudinal changes. Lentic and lotic ecosystems differ widely in biotic (prey and fish communities) and abiotic (habitat complexity and physical connectivity) conditions^15,35,46^, which may favour trophic-niche divergence, and thus the dietary habits of fish usually change across ecosystem type^31,47^. The linkage between latitude and piscivory appears to be complex and different mechanisms can potentially strengthen the latitudinal gradient in piscivory. Thus, it is possible that a low availability of alternative prey (other than fish) promotes piscivory in lacustrine populations at higher latitudes, in line with the aforementioned view that lacustrine systems commonly support lower benthic macroinvertebrate production compared to riverine systems^35^. However, ecosystems were not represented equally across the latitudinal gradient in this study, a bias that might limit the analyses of latitudinal gradients in piscivory. Furthermore, this study suggests that fish community composition and ecosystem type can be more influential than latitude. Yet latitude, and hence temperature gradients, may partially explain variation in the prevalence of piscivory in other model organisms^6,7,16,17^, but highly dependent on fish community [i.e. fish species richness and the role (prey, competitors or predators) displayed by sympatric fish species] as indicated in the current study.

As already mentioned, there are some limitations and considerations that need to be acknowledged regarding the conclusions of this study. Firstly, this study focussed on a single, but globally distributed, aquatic predator, and studies beyond the model organism are needed to corroborate or refute general conclusions about piscivory. It is likely that similar responses will be observed in closely related species (i.e. salmonids), but it is unlikely that the conclusions from this study can be broadly extrapolated to distantly related taxa^32^. Secondly, the data set used in this study may suffer limitations from an over-representation of data points at higher latitudes due to possible biases caused by uneven sampling effort across the different territories. From another standpoint, the unequal sample sizes across the latitudinal gradient illustrate the need for more investigations within the relatively unstudied parts of the brown trout’s native range. Thirdly, this study included only native populations, which imposes a limitation on the extrapolation of the conclusions of this study to non-native territories (i.e. the role of invasiveness), which may be a promising avenue for future research in salmonids. Fourthly, ecosystem size and the abundance and body sizes of fish prey assemblages have repeatedly emerged as an important determinant of piscivory^5,8,9,48^. Although these variables were considered during the conception of this study, such information was missing in most data sources and thus prevented their inclusion. However, these variables should be taken into consideration in future studies exploring variation in the piscivory of animals. Finally, because the size-structure of brown trout populations was not similar among ecosystem types, it is possible that cross-ecosystem differences in piscivory may be biased by larger individuals in lentic ecosystems. However, it is also possible that predator body size is a key driver of piscivory in the early development of some species, but a consequence for adult individuals. Notwithstanding, the current study represents an innovative attempt, based on a multifactor approach, to identify possible synergies (i.e. combined effects of several factors) that influence piscivory, and provides novel insights into the importance of ecosystem type, site-specific fish communities, body size and temperature-related geographical (latitude rather than elevation) variables as drivers of variations in the prevalence of piscivory in brown trout populations.

To conclude, this study provides empirical support for the notion that piscivory can be explained by geographic (higher in lentic than lotic ecosystems) and latitudinal (increasing with latitude in lacustrine populations) gradients interacting with fish community configuration and the size-structure (ontogeny) of brown trout populations. Such variations in piscivory may have profound implications for the understanding of population dynamics and food-web stability^49,50^. For example, it is reasonable to posit that trophic cascades (top-down mechanisms) mediated by apex predators (here brown trout) are more noticeable in lentic compared to lotic ecosystems and might increase with latitude. Thus, the results of this study should also be of interest to aquatic ecologists, as predator–prey interactions have important ecological implications for food-web structure.

## Methods

### Data compiling (systematic reviews)

I carried out a systematic review of peer-reviewed publications aiming to reach a broad understanding of what drives piscivory in brown trout (i.e. the percentage of individuals in a population that are piscivorous). Data compiling followed explicit guidelines and standards for conducting meta-analyses to (i) ensure good practices in the searching, curation and evaluation of data, (ii) ensure reproducibility, and (iii) reduce bias^51–53^. The data were found by literature searches in the Web of Science^®^ using the key term “brown trout” in combination with “feeding”, “piscivory” and “*Salmo trutta*”, i.e. the keyword sequence: *TITLE*: (*brown trout*)* AND TITLE*: (*feeding*)* OR TITLE*: (*piscivory*)* AND TOPIC*: (*Salmo trutta*). In summary, the literature review consisted of published data (peer-reviewed papers) on piscivory by brown trout across three ecosystem configurations (riverine, lacustrine and marine) from 99 papers from the Web of Science Core Collection. It should be kept in mind that searches based solely on online citation databases, such as the Web of Science, may be incomplete because they selectively catalogue citations according to a predefined list of journals or publishers^54^, and the searches may derive from how they are accessed by researchers instead of from what they contain^55^. Therefore, it is good practice to use more than one search engine for meta-analyses^54,55^. In this study, the literature search was extended to Google Scholar using the same keyword sequence used in the Web of Science. Search results in Google Scholar were performed at title level (i.e. only the title of each document was searched for the specified terms) and excluded grey literature. In addition to the literature search from the Web of Science Core Collection, another 120 relevant primary studies from Google Scholar (only published articles) were included. Next, the compiled articles (*n* = 219) were reviewed and only articles that met all of the following criteria were included:

1. Population-level differences in the prevalence of piscivory (i.e. frequency of occurrence, %) based on the presence–absence method; when information was only provided in terms of counts (i.e. the number of piscivorous individuals), piscivory was calculated as frequency of occurrence. The presence–absence method is the simplest and least time-consuming approach, providing reliable documentation of the diversity and prevalence of prey utilised by predator populations^56^.
2. Detailed diet categories or at least a clear category indicating the prevalence of piscivory or the prevalence of trophically transmitted parasites via fish prey (i.e. parasites that are host-specific for prey species)^57^.
3. Brown trout was considered native in the system. That is, non-native populations were dismissed to avoid invasiveness of the species and hence confounding effects caused by a higher degree of piscivory in non-native areas where the native fish fauna has not coevolved with brown trout^58^, which could complicate the evaluation of the drivers responsible for variations in the prevalence of piscivory across ecosystems.
4. Field studies (not laboratory or experimental studies).
5. Ecosystem type, fish community configuration, brown trout body size, elevation and latitude reported. Concerning ecosystem type, compiled studies were sorted in three groups: riverine (*n* = 42), lacustrine (*n* = 53) and marine (*n* = 25, i.e. studies restricted only to marine feeding in anadromous populations). Using previously described categories^5^, each population was assigned to a fish community configuration on the basis of fish species richness, namely (i) one-species systems (*n* = 14, i.e. brown trout was the only fish species present; any piscivory was cannibalistic), (ii) two-species systems (*n* = 25, i.e. brown trout and one other fish species), (iii) three-species systems (*n* = 31, i.e. brown trout and two other fish species), and (iv) multi-species systems (*n* = 50, i.e. brown trout and three or more other fish species). Body size information referred to the observed maximum length, as average length was only available in 34 out of 120 records (see Supplementary Appendix S5 for further information about brown trout body size distributions). However, I also conducted sensitivity analyses with average length (see “Modelling approach” section).

After this first selection, data were standardised to (1) exclude first-feeding fry; (2) consider lakes or localities within the same river but with different fish communities as different records; and (3) sum data from the same sampling site but over several months as a single value. When studies included data published in another paper, data were taken from the primary source reporting the most complete information.

**Figure 3:**
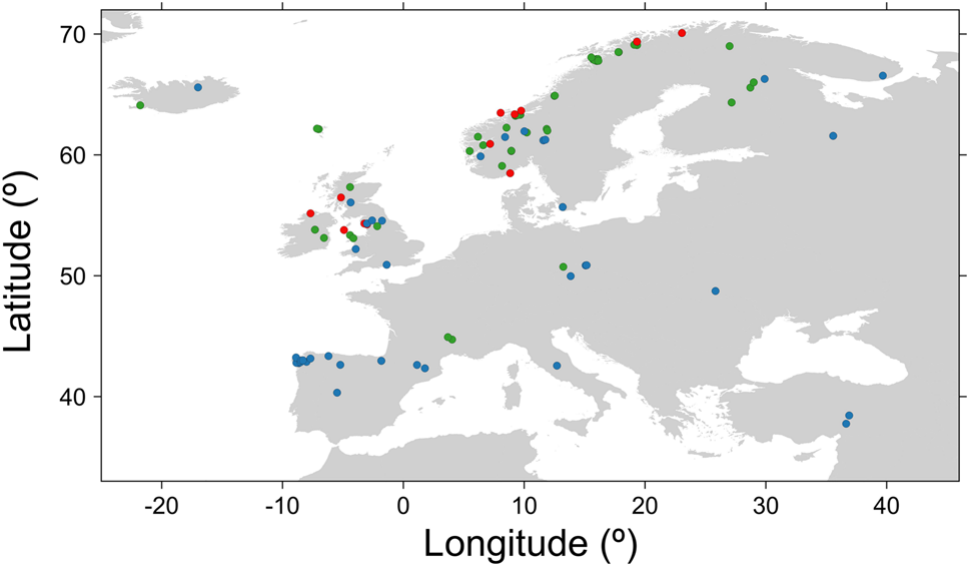
Map showing the location of the sampling sites used in this study. Blue circles = riverine populations, green circles = lacustrine populations and red circles = marine (anadromous with marine feeding) populations.

In line with the above-described selection criteria, a total of 66 studies (some of them with different records) were judged to be satisfactory to be included in the dataset. Thus, the final database contained a total of 120 populations spanning 34° S to 70° N, which covers a substantial part of the species’ natural geographical range and includes mostly Europe, Faroe Islands and Iceland (Fig. 3; the 66 data sources used in this study are listed in Supplementary Appendix S6). The different studies vary markedly in scope, sample size and fish community configuration. Because of the high species richness scores in the marine ecosystems^26,27^, these ecosystems only included multi-species systems. Except for one-species systems (only shaped by intraspecific competition), trophic relationships can be diverse as fish species may be prey [e.g. three-spined stickleback *Gasterosteus aculeatus* L. and minnow *Phoxinus phoxinus* (L. 1758)], potential competitors [e.g. Arctic charr *Salvelinus alpinus* (L.) and brook trout *Salvelinus fontinalis* (Mitchill, 1814)] or even predators (e.g. pike *Esox lucius* L.). Sympatric fish species were assigned as prey, competitors or predators on the basis of knowledge about predator–prey interactions^5,12,36,59^. *Salvelinus* species can be both prey for and predators of brown trout depending on size^5,33,34^. However, when brown trout and *Salvelinus* species coexist, the prevalence of piscivory of brown trout is usually very low^5,34^. Thus, two-species systems with brown trout and Arctic charr or brook trout were considered as fish competitor systems mediated by intraguild predation. In line with this criterion, each population was also assigned according to: (i) cannibalism (*n* = 14, i.e. one-species systems), (ii) fish prey systems (*n* = 30, i.e. only fish prey in the fish community configuration), (iii) fish competitor systems (*n* = 15, i.e. only potential fish competitors in the fish community configuration; representing the potential for intraguild predation), (iv) two-prey systems (*n* = 30, i.e. fish prey and potential competitors in the fish community configuration), and (v) three-prey systems (*n* = 31, i.e. fish prey, competitors and predators in the fish community configuration).

When geographical coordinates (i.e. latitude and elevation) of the study area were not provided in a literature source, the coordinates were digitalised based upon assessments of location information or maps provided in the source. Pairwise differences in piscivory within- and between-ecosystem ecosystem types were tested through non-parametric Mann–Whitney–Wilcoxon tests (Shapiro–Wilk tests indicated non-normality in the data) with significance levels adjusted by applying the Bonferroni method. A significance level of *P* = 0.05 was used in all analyses.

### Meta-analysis (meta-regression)

When combining the results from different studies (i.e. meta-analysis), there is a need to identify sources of heterogeneity and control effect sizes (i.e. the causes of variation among study outcomes) by using heterogeneity tests and meta-regressions^52,53^. Thus, the statistical procedures carried out in this study covered effect-size calculation, heterogeneity analysis and weighting following previously described methods^53^. Using the R “metafor” package^60^, the effect size (i.e. the magnitude of a relationship between piscivory and sample size) was calculated using mixed-effects models to control for variation (in the methods and/or sample characteristics) attributable to different studies^52,61^. The trim and fill method^62^ implemented for funnel plots was used to identify publication bias and between-study heterogeneity (Supplementary Appendix S7).

### Modelling approach

Prior to modelling, variance inflation factors (VIF) were used to detect multicollinearity (correlation between predictors) between latitude and the other quantitative covariates (fish length, elevation, annual mean temperature, temperature annual range, temperature seasonality, temperature of warmest month and mean temperature of warmest quarter)^24,63^. Zuur et al. recommended VIF < 3 as an indicator of low evidence for collinearity^64^. Accordingly, temperature variables were dropped in subsequent analyses (Supplementary Appendix S8). The final dataset consisted of piscivory, ecosystem type, fish community type, fish prey type, latitude, elevation and brown trout body size.

Because data did not meet normality and hence the assumptions for linear regression models^65^, I explored the response of piscivory along quantitative variables (latitude, elevation and brown trout size) as smoother terms using generalised additive models (GAMs) in the R “mgcv” package^66^. Models were fitted using the REML method (automatic estimation of the amount of smoothing). This analysis allowed non-linear trends in piscivory along predictors to be disentangled and the assumption that an underlying linear correlation exists between two variables to be avoided^67^.

Next, a multiple regression approach was used to explore combined factors that can determine patterns in the prevalence of piscivory using generalised additive mixed models (GAMMs) in the R “mgcv” package. To prevent results being affected by publication bias and between-study heterogeneity as indicated by meta-regression (Supplementary Appendix S7), the modelling approach was performed with study (i.e. the data sources used in this study) as a random effect. This allowed the variation attributable to different studies being conducted by different researchers and with different sampling methods to be controlled^53,65^, and also possible variation in piscivory resulting from variables not considered in the current study, such as inherent feeding traits linked to, for example, populations and/or phylogeographical lineages of the model organism. Because smoothers in GAMMs are meant for continuous (not categorical) explanatory variables, I included interaction terms between continuous (latitude, elevation and body size) and categorical variables (ecosystem type, fish community type and fish prey type) as smoothers. The full model then consisted of three predictor variables and ten smoother terms [~ latitude + elevation + body size + s(latitude − ecosystem type) + s(latitude − fish community type) + s(latitude − fish prey type) + s(elevation − ecosystem type) + s(elevation − fish community type) + s(elevation − fish prey type) + s(body size − ecosystem type) + s(body size − fish community type) + s(body size − fish prey type) + s(latitude − ecosystem type × fish community type)] with study as random factor. Using a model selection method^68^, I ranked the candidate models according to the Akaike information criterion (AICc; the best model being the one with the lowest AICc value). Model selection was done by model comparison using the *dredge* (Automated Model Selection) function in the R “MuMIn” package^69^. This procedure generated a set of models with combinations (subsets) of fixed effect terms in the full model ranked according to AICc values. Note that models with AICc values within 1–2 units of the best model also have substantial support^68^. The significance table of the final selected models was extracted using the *summary* function and residuals were visually inspected for deviations from normality and heteroscedasticity. Finally, I also produced models including only: (i) multispecies systems and (ii) sampling events that provided average length (Supplementary Appendix S6). This sensitivity analysis enabled me to (i) control possible imbalanced data compiling in fish community type among ecosystem types and (ii) use a likely more informative variable of population size-structure than maximum length, respectively.

## Supplementary information

Supplementary Information. (PDF 937 kb)

## Data Availability

The dataset generated and analysed during the current study is available in the Supplementary Appendix S6.
