# Supplementary material for: Drivers of piscivory in a globally distributed aquatic predator (brown trout): a meta-analysis
Source: Sci Rep. 2020 Jul 9;10:11258. doi: 10.1038/s41598-020-68207-8 (PMC7347837; doi:10.1038/s41598-020-68207-8)
Supplement: Supplementary file 1 — Supplementary Information. (PDF 937 kb) [file 41598_2020_68207_MOESM1_ESM.pdf]

Drivers of piscivory in a globally distributed aquatic predator (brown trout): A meta-analysis

Javier Sánchez-Hernández*

Área de Biodiversidad y Conservación, Departamento de Biología y Geología, Física y Química Inorgánica, Universidad Rey Juan Carlos, Móstoles, Madrid

*Author for correspondence (Tel.: +34 630 156 186; E-mail: jav.sanchez.hernandez@gmail.com; ORCID id: 0000-0001-9684-4774).

Appendix S1: Model selection table (*GAMMs*) including the 10 best model simulations. Note that models with AIC values within 1–2 units of the best model also have substantial support (Burnham & Anderson 2002) and marked in grey. The best model is in bold.

Burnham, K.P. & Anderson, D.R. (Editors). 2002. Model selection and multimodel inference: a practical information-theoretical approach. 2nd ed. Springer-Verlag. New York.

A) Models including all dataset: Ecosystem type (ET), fish community type (FCT), fish prey type (FPT).

Model	Intercept	Predictor variables			Smooth terms										Model statistics				
		Elevation	Latitude	Body size	Latitude–ET*FCT	Elevation–ET	Elevation–FCT	Elevation–FPT	Latitude–ET	Latitude–FCT	Latitude–FPT	Body size–ET	Body size–FCT	Body size–FPT	df	logLik	AICc	delta	weight
**1**	**45.380**	**—**	**-1.172**	**0.061**	**+**	**—**	**—**	**—**	**—**	**—**	**—**	**—**	**—**	**+**	**11**	**-413.806**	**852.544**	**0**	**0.399**
2	42.544	0.004	-1.146	0.061	+	—	—	—	—	—	—	—	—	+	12	-413.353	854.212	1.667	0.173
3	42.061	—	-1.057	0.045	+	—	—	—	—	—	—	—	—	—	8	-419.187	855.921	3.377	0.074
4	-18.305	—	—	0.054	+	—	—	—	—	—	—	—	—	+	10	-417.667	857.751	5.206	0.030
5	39.092	0.003	-1.026	0.046	+	—	—	—	—	—	—	—	—	—	9	-418.951	857.858	5.314	0.028
6	51.740	—	-1.301	0.061	+	—	—	+	—	—	—	—	—	+	14	-412.835	858.498	5.954	0.020
7	-20.238	0.005	—	0.055	+	—	—	—	—	—	—	—	—	+	11	-416.968	858.869	6.324	0.017
8	-15.827	—	—	0.040	+	—	—	—	—	—	—	—	—	—	7	-421.854	858.900	6.355	0.017
9	51.771	—	-1.315	0.063	+	+	—	—	—	—	—	—	—	+	14	-413.047	858.921	6.377	0.016
10	35.862	—	-0.913	0.038	+	—	—	—	—	—	—	—	+	—	11	-417.070	859.073	6.528	0.015

B) Sensitivity analyses (dataset with only mean body size): Ecosystem type (ET), fish community type (FCT), fish prey type (FPT).

Model	Intercept	Predictor variables			Smooth terms						Model statistics				
		Elevation	Latitude	Body size	Latitude–ET*FPT	Elevation–ET	Elevation–FPT	Latitude–ET	Latitude–FPT	Body size–FPT	df	logLik	AICc	delta	weight
**1**	**-9.048**	**—**	**—**	**—**	**+**	**—**	**—**	**—**	**—**	**—**	**6**	**-152.807**	**320.725**	**0**	**0.301**
2	56.208	-0.086	—	—	—	—	—	—	—	—	4	-156.248	321.875	1.149	0.169
3	28.073	-0.086	—	0.098	—	—	—	—	—	—	5	-155.475	323.093	2.368	0.092
4	0.093	-0.022	—	—	+	—	—	—	—	—	7	-152.577	323.461	2.736	0.077
5	41.618	—	-0.821	—	+	—	—	—	—	—	7	-152.672	323.651	2.926	0.070
6	-11.777	-0.077	1.048	—	—	—	—	—	—	—	5	-155.885	323.914	3.188	0.061
7	-10.172	—	—	0.006	+	—	—	—	—	—	7	-152.804	323.915	3.190	0.061
8	-31.333	-0.076	0.926	0.094	—	—	—	—	—	—	6	-155.131	325.372	4.647	0.029
9	43.070	—	—	—	—	—	—	—	—	—	3	-159.813	326.426	5.700	0.017
10	-94.978	—	2.180	—	—	—	—	—	—	—	4	-158.575	326.530	5.805	0.017

C) Sensitivity analyses (dataset with only including multispecies systems): Ecosystem type (ET), fish community type (FCT), fish prey type (FPT).

Model	Intercept	Predictor variables			Smooth terms						Model statistics				
		Elevation	Latitude	Body size	Latitude–ET*FPT	Elevation–ET	Elevation–FPT	Latitude–ET	Latitude–FPT	Body size–FPT	df	logLik	AICc	delta	weight
**1**	**-17.506**	**—**	**—**	**0.077**	**+**	**—**	**—**	**—**	**—**	**—**	**7**	**-144.028**	**306.364**	**0**	**0.534**
2	-20.990	0.009	—	0.072	+	—	—	—	—	—	8	-143.652	309.064	2.699	0.139
3	-25.437	—	0.147	0.078	+	—	—	—	—	—	8	-143.995	309.750	3.386	0.098
4	-109.370	—	1.726	0.099	—	—	—	—	—	—	5	-149.415	310.973	4.609	0.053
5	-30.331	—	—	0.123	+	—	—	—	—	+	10	-140.858	311.282	4.918	0.046
6	-53.491	0.015	0.563	0.073	+	—	—	—	—	—	9	-143.290	312.081	5.716	0.031
7	-132.896	0.015	2.066	0.100	—	—	—	—	—	—	6	-148.686	312.483	6.119	0.025
8	-161.182	—	2.567	0.096	—	—	—	+	—	—	8	-145.427	312.614	6.250	0.023
9	-158.017	—	2.539	0.093	—	—	—	—	+	—	8	-145.821	313.401	7.037	0.016
10	-34.108	0.009	—	0.117	+	—	—	—	—	+	11	-140.386	314.772	8.408	0.008

-Appendix S2: Residual structure (GAMMs).

QQ-plot was used to assess normality (if the points are in a line, normality can be assumed). Heteroscedasticity (the absence of trend in the residuals) was evaluated plotting predicted values versus residuals.

Figure S2A. Models including all dataset.

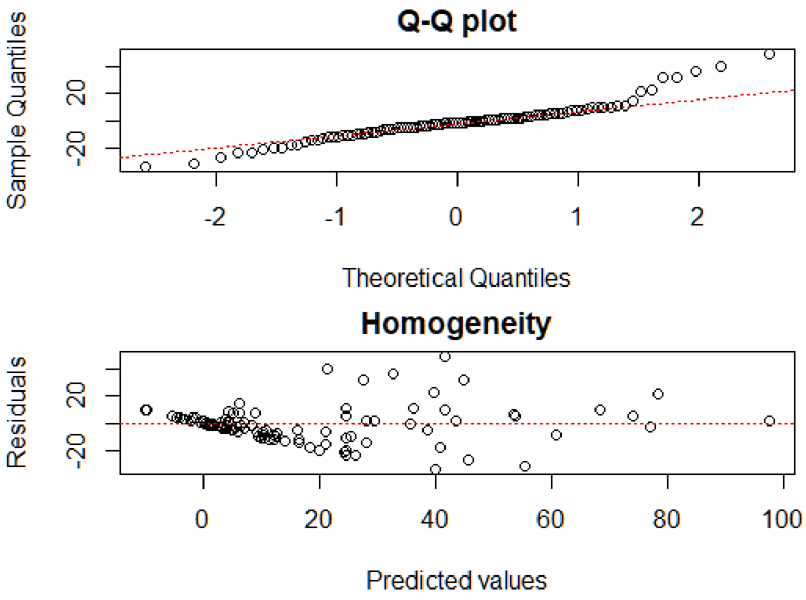

Figure S2B. Sensitivity analyses (dataset with only mean body size).

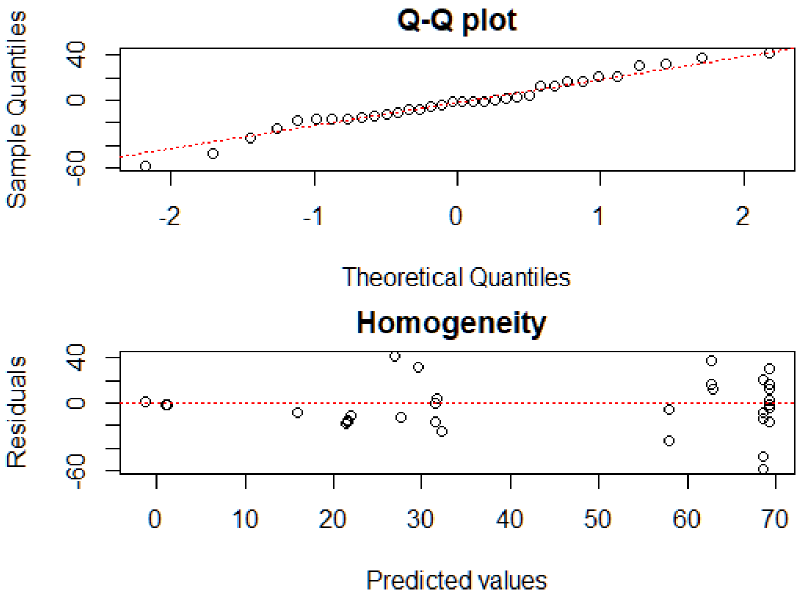

Figure S2C. Sensitivity analyses (dataset with only including multispecies systems).

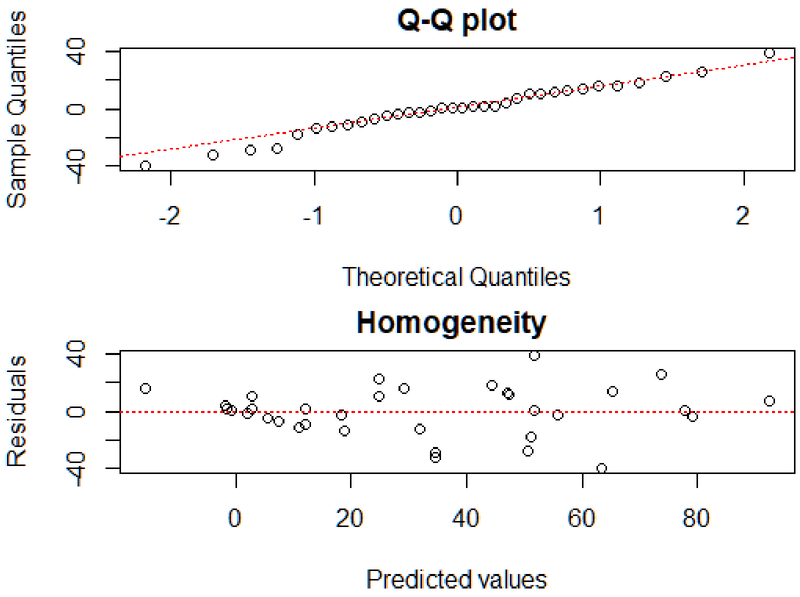

-Appendix S3: Within-ecosystem and between-ecosystem pairwise comparisons of pisvivory (prevalence, %).

Shapiro–Wilk tests indicated non-normality in the data, and thus pairwise comparison was carried out using a non-parametric test (Mann-Whitney-Wilcoxon). Significance levels were adjusted by applying the Bonferroni method, which are shown in brackets. Significant values are marked in bold. Marine ecosystem only included multi-species communities.

A) Sampling size used in pairwise comparisons and correlations (Appendix S4)

Fish community type	Fish prey type	Ecosystem type		
		River	Lake	Marine
One-species	Cannibalism	5	9	-
Two-species	Prey	26	4	-
	Competitor	4	11	-
Three-species	Prey+Competitior	7	23	-
Multi-species	Prey+Competitior+Predator	-	6	25

B) Within-ecosystem pairwise comparisons of piscivory (%) among brown trout populations:

	Riverine (*n* = 42)	Lacustrine (*n* = 53)
Fish community type		
One-species *versus* two-species systems	W = 7.5, **$P = 0.014$** ($P = 0.086$)	W = 6.5, **$P < 0.001$** (**$P = 0.002$**)
One-species *versus* three-species systems	W = 7.5, **$P = 0.018$** ($P = 0.109$)	W = 16.5, **$P < 0.001$** (**$P = 0.003$**)
One-species *versus* multi-species systems	W = 12.5, **$P = 0.028$** ($P = 0.166$)	W = 5.0, **$P < 0.001$** (**$P = 0.001$**)
Two-species systems *versus* three-species systems	W = 66.5, $P = 1.000$ **028** ($P = 1.000$)	W = 80.0, $P = 0.068$ ($P = 0.407$)
Two-species systems *versus* multi-species systems	W = 90.5, $P = 0.754$ ($P = 1.000$)	W = 2.0, **$P < 0.001$** (**$P < 0.001$**)
Three-species systems *versus* multi-species systems	W = 84.0, $P = 0.717$ ($P = 1.000$)	W = 45.5, **$P = 0.008$** (**$P = 0.049$**)
Fish prey type		
Cannibalism *versus* fish prey	W = 15.0, **$P = 0.006$** (**$P = 0.039$**)	$W = 5.0$, **$P = 0.033$** (**$P = 0.033$**)
Cannibalism *versus* fish competitor	W = 2.5, **$P = 0.042$** ($P = 0.250$)	$W = 6.5$, **$P < 0.001$** (**$P = 0.008$**)
Cannibalism *versus* fish prey+competitor	W = 10.0, $P = 0.136$ ($P = 0.814$)	$W = 16.5$, **$P < 0.001$** (**$P = 0.002$**)
Cannibalism *versus* fish prey+competitor+predator	—	$W = 5.0$, **$P < 0.001$** (**$P = 0.010$**)
Fish prey *versus* fish competitor	W =40.0, $P = 0.480$ ($P = 1.000$)	$W = 44$, **$P = 0.001$** (**$P = 0.015$**)
Fish prey *versus* fish prey+competitor	W = 122.5, $P = 0.166$ ($P = 0.997$)	$W = 59$, $P = 0.393$ ($P = 1.000$)
Fish prey *versus* fish prey+competitor+predator	—	$W = 7.0$, $P = 0.352$ ($P = 1.000$)
Fish competitor *versus* fish prey+competitor	W = 21.0, $P= 0.198$ ($P = 1.000$)	$W = 56.0$, **$P = 0.010$** ($P = 0.099$)
Fish competitor *versus* fish prey+competitor+predator	—	$W = 5.0$, **$P < 0.001$** (**$P = 0.002$**)
Fish prey+competitor *versus* fish prey+competitor+predator	—	$W = 26.5$, **$P = 0.024$** ($P = 0.236$)

C) Between-ecosystem pairwise comparisons of piscivory (%) among brown trout populations:

	Riverine *versus* lacustrine	Riverine *versus* marine	Lacustrine *versus* marine
Fish community type			
One-species	$W = 17.5$, $P = 0.325$ ($P = 0.320$)	—	—
Two-species	$W = 56.5$, $P = 0.252$ ($P = 0.250$)	—	—
Three-species	$W = 52.5$, **$P = 0.018$** (**$P = 0.018$**)	—	—
Multi-species	$W = 5$, **$P < 0.001$** (**$P < 0.001$**)	$W = 10$, **$P < 0.001$** (**$P < 0.001$**)	$W = 125.5$, $P = 0.693$ ($P = 1.000$)
Fish prey type			
Cannibalism	$W = 17.5$, $P = 0.325$ ($P = 0.320$)	—	—
Fish prey	$W = 5.0$, **$P = 0.002$** (**$P = 0.002$**)	—	—
Fish competitor	$W = 24.5$, $P = 0.794$ ($P = 0.790$)	—	—
Fish prey+competitor	$W = 23.0$, **$P = 0.005$** (**$P = 0.005$**)	—	—
Fish prey+competitor+predator	—	—	$W = 75.5$, $P = 1.000$ ($P = 1.000$)

-Appendix S4: Response of piscivory (%) along predictors (latitude, and elevation and maximum brown trout length) using generalised additive models (GAMs).

A) Riverine systems:

Riverine systems did not include three-prey (prey, competitors and predators) systems and piscivory = 0% in one-species systems. Edf = estimated degree of freedom for smooth terms are shown. ***P < 0.001, **P < 0.01 and *P < 0.05.

	Constant	Smooth terms (Edf)			Model statistics		
		Latitude	Elevation	Body size	Observations	Adjusted R^2	Deviance explained (%)
Latitude							
Total	3.049***	1.000	—	—	42	-0.021	0.42
Fish community = two-species systems	3.197**	1.000	—	—	12	0.227	29.7
Fish community = three-species systems	3.936	1.000	—	—	11	0.040	13.6
Fish community = multi-species systems	3.314*	1.000	—	—	14	-0.080	0.29
Fish type = prey fish system	3.405***	1.000	—	—	26	-0.014	2.61
Fish type = fish competitor system	7.120	1.946	—	—	4	0.944	98
Fish type = two-prey (prey and competitors) system	1.579	1.000	—	—	7	0.356	46.3
Elevation							
Total	3.049***	—	1.000	—	42	-0.007	1.69
Fish community = two-species systems	3.197*	—	1.000	—	12	-0.071	2.62
Fish community = three-species systems	3.936	—	1.000	—	11	0.028	12.5
Fish community = multi-species systems	3.314*	—	1.000	—	14	-0.082	0.13
Fish type = prey fish system	3.405***	—	1.000	—	26	-0.041	0.08
Fish type = fish competitor system	7.120	—	1.000	—	4	0.131	42.1
Fish type = two-prey (prey and competitors) system	1.579	—	1.000	—	7	-0.130	5.84
Body size							
Total	3.201***	—	—	1.566	40	0.068	10.6
Fish community = two-species systems	3.385**	—	—	1.000	12	0.175	25
Fish community = three-species systems	3.828	—	—	1.304	11	-0.063	7.71
Fish community = multi-species systems	3.757*	—	—	1.000	12	0.033	12.1
Fish type = prey fish system	3.102***	—	—	1.000	24	0.027	6.9
Fish type = fish competitor system	6.260	—	—	1.000	4	-0.012	32.6
Fish type = two-prey (prey and competitors) system	4.044	—	—	1.000	7	-0.175	2.04

B) <u>Lacustrine systems</u>:

Edf = estimated degree of freedom for smooth terms are shown. ****P* < 0.001, ***P* < 0.01 and **P* < 0.05.

	Constant	Smooth terms (Edf)			Model statistics		
		Latitude	Elevation	Body size	Observations	Adjusted R^2	Deviance explained (%)
Latitude							
Total	20.908***	1.000	—	—	53	-0.005	1.4
Fish community = one-species systems	0.078	1.000	—	—	9	0.040	16
Fish community = two-species systems	5.994**	1.870	—	—	13	0.337	44
Fish community = three-species systems	23.191***	1.311	—	—	20	0.010	7.8
Fish community = multi-species systems	51.424***	1.000*	—	—	11	0.389	45
Fish type = prey fish system	35.912**	1.000**	—	—	4	0.973	98.2
Fish type = fish competitor system	3.512**	4.038*	—	—	11	0.708	82.6
Fish type = two-prey (prey and competitors) system	25.474***	1.000	—	—	23	0.009	5.45
Fish type = three-prey (prey, competitors and predators) system	56.538***	1.000*	—	—	6	0.678	74.2
Elevation							
Total	20.908***	—	1.000	—	53	-0.001	1.8
Fish community = one-species systems	0.078	—	1.000	—	9	0.011	13.5
Fish community = two-species systems	5.994*	—	1.000	—	13	-0.013	7.1
Fish community = three-species systems	23.191***	—	1.000	—	20	0.032	8.3
Fish community = multi-species systems	51.424***	—	1.000	—	11	-0.057	4.84
Fish type = prey fish system	35.912	—	1.972	—	4	0.917	97.2
Fish type = fish competitor system	3.512**	—	1.000	—	11	-0.088	2.07
Fish type = two-prey (prey and competitors) system	25.474***	—	1.000	—	23	0.028	7.18
Fish type = three-prey (prey, competitors and predators) system	56.538*	—	1.000	—	6	-0.250	0.01
Body size							
Total	19.346***	—	—	1.359***	50	0.262	28.3
Fish community = one-species systems	0.078	—	—	1.000	9	-0.143	0.01
Fish community = two-species systems	6.652*	—	—	1.259	13	-0.059	5.2
Fish community = three-species systems	23.293***	—	—	1.251*	20	0.224	27.5
Fish community = multi-species systems	51.781**	—	—	1.000	8	0.055	19
Fish type = prey fish system	28.733	—	—	1.620	3	0.562	91.7
Fish type = fish competitor system	6.296	—	—	1.000	11	-0.098	1.14
Fish type = two-prey (prey and competitors) system	28.659***	—	—	1.000	23	0.130	16.9
Fish type = three-prey (prey, competitors and predators) system	37.997	—	—	1.683	4	0.810	91.7

Figure S4A. Residual structure (GAMs) for models with statistically significant smooth terms according to the above table.

QQ-plot was used to assess normality (if the points are in a line, normality can be assumed). Heteroscedasticity (the absence of trend in the residuals) was evaluated plotting predicted values versus residuals.

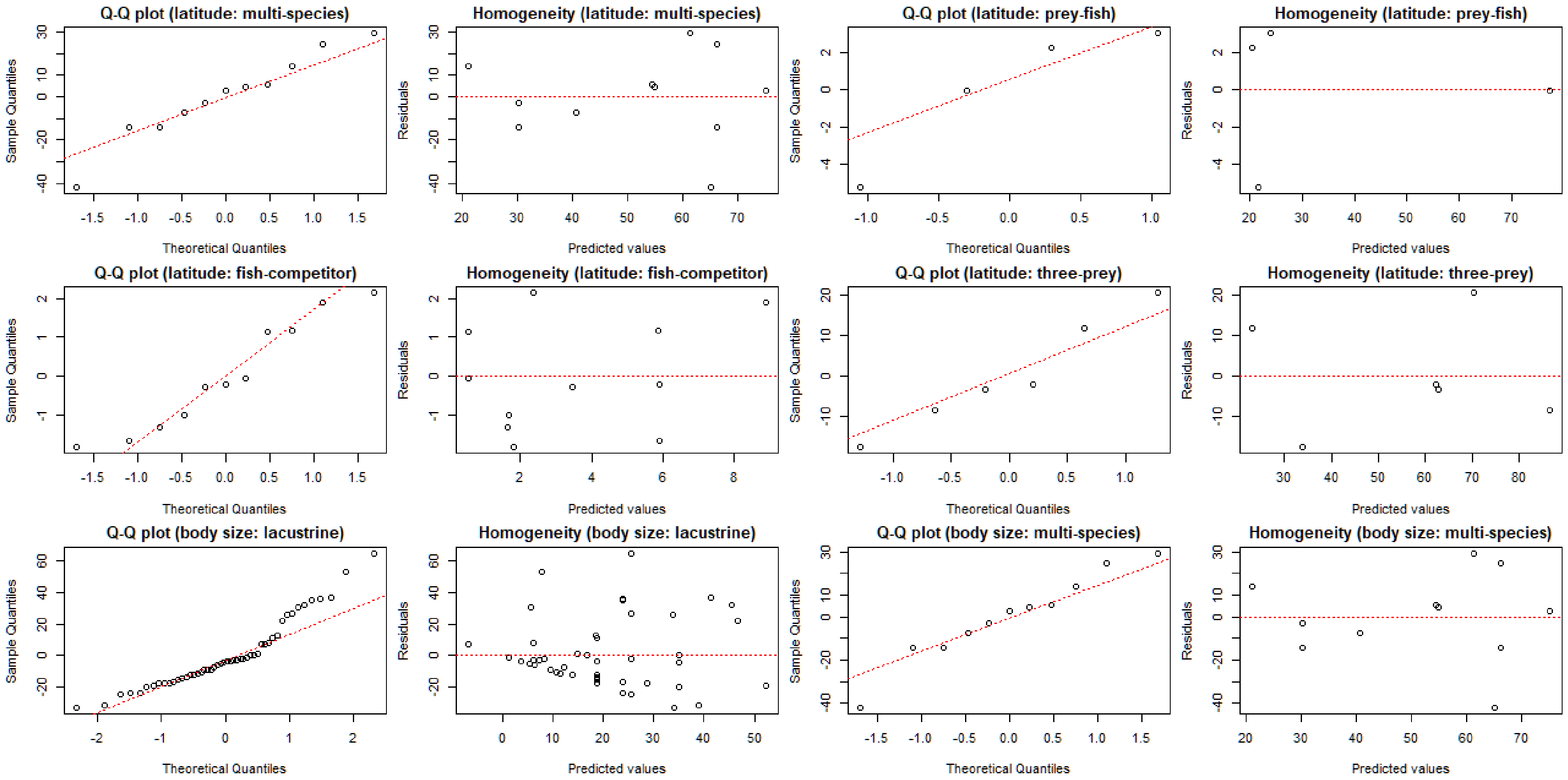

C) <u>Marine systems</u>:

Marine systems only included multi-species systems with three-prey (prey, competitors and predators) systems. *Marine ecosystems included data records at sea level. Edf = estimated degree of freedom for smooth terms are shown. ****P* < 0.001, ***P* < 0.01 and **P* < 0.05.

	Constant	Smooth terms (Edf)			Model statistics		
		Latitude	Elevation	Body size	Observations	Adjusted R^2	Deviance explained (%)
Latitude							
Fish community = multi-species systems	55.756***	3.398	—	—	25	0.309	40.7
Elevation*							
Fish community = multi-species systems	—	—	—	—	—	—	—
Body size							
Fish community = multi-species systems	51.191***	—	—	1.950	12	0.389	49.7

Figure S4B. Prevalence of piscivory (%) along covariates (latitude, elevation and maximum body size). Data are displayed for type of ecosystem (riverine, lacustrine and marine) and fish prey type (cannibalism, fish prey, fish competitor, fish prey and competitors, and fish prey, competitors and predators). Fitted lines with 95% confidence intervals are only shown for statistically significant trends (see above).

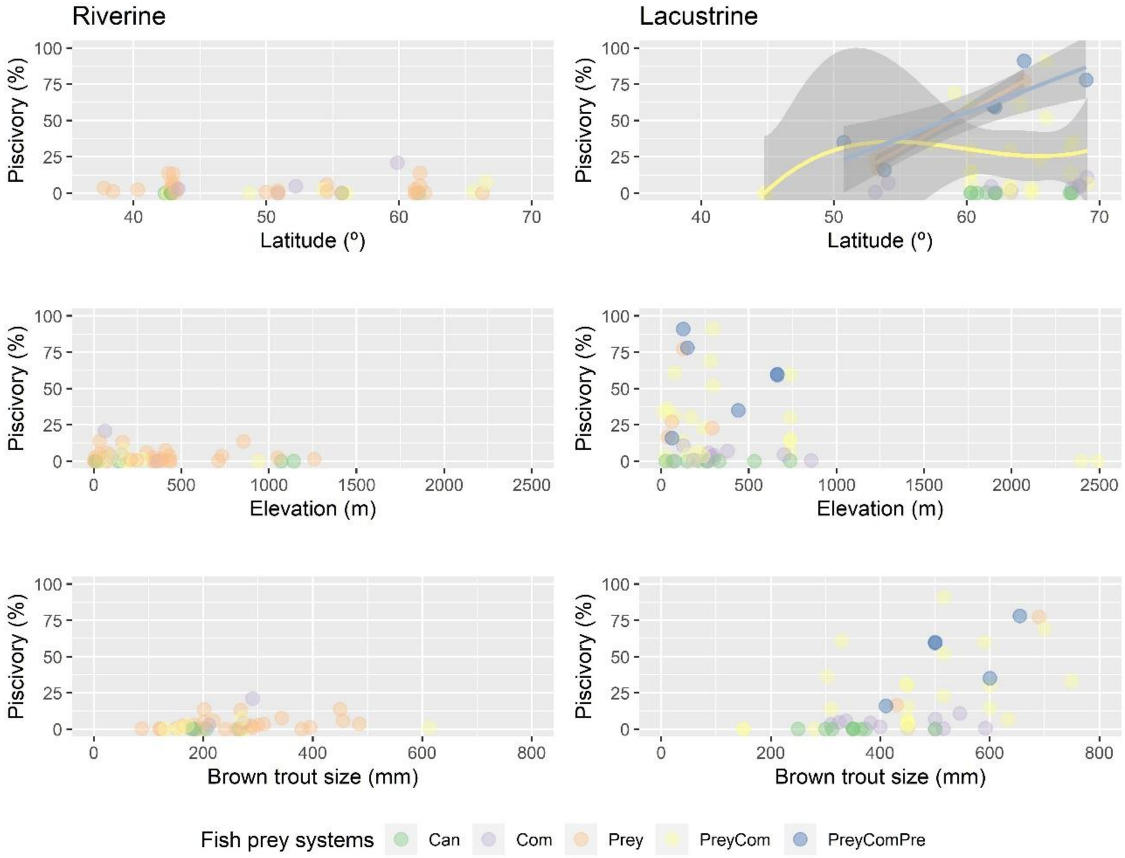

-**Appendix S5:** Brown trout body size distributions (maximum, minimum and mean body size).

A) Between-ecosystem pairwise comparisons:

Shapiro–Wilk tests indicated non-normality in the data, and thus pairwise comparison was carried out using a non-parametric test (Mann-Whitney-Wilcoxon). Significance levels were adjusted by applying the Bonferroni method, which are shown in brackets. Significant values are marked in bold.

	Mean (mm) ± SE	Range (mm)	Pairwise comparison		
			Riverine *vs* lacustrine	Riverine *vs.* marine	Lacustrine *vs.* marine
Maximum body size					
Riverine	253.4 ± 17.47	88-613	*W* = 255.5, ***P* < 0.001** (***P* < 0.001**)	*W* = 49, ***P* < 0.001** (***P* < 0.001**)	*W* = 216.5, *P* = 0.138 (*P* = 0.929)
Lacustrine	446.6 ± 18.93	150-750			
Marine	517.1 ± 47.81	225-725			
Minimum body size					
Riverine	84.9 ± 10.51	25-380	*W* = 424, ***P* < 0.001** (***P* < 0.001**)	*W* = 25, ***P* < 0.001** (***P* < 0.001**)	*W* = 76, ***P* < 0.001** (***P* = 0.006**)
Lacustrine	131.3 ± 6.96	48-240			
Marine	223.4 ± 19.48	130-346			
Mean body size					
Riverine	120.8	—			
Lacustrine	231.6 ± 17.21	158.7-388	—	—	*W* = 35, ***P* < 0.001** (***P* = 0.001**)
Marine	315.8 ± 11.83	248-433			

Figure S5A. Violin plots (box plots with the probability density) showing body size distributions of brown trout according to ecosystem type (1 = riverine, 2 = lacustrine, 3 = marine). The boxplot within each violin plot indicates the median and the interquartile range with the 95% confidence interval for the median. Left part = maximum body size. Right part = mean body size.

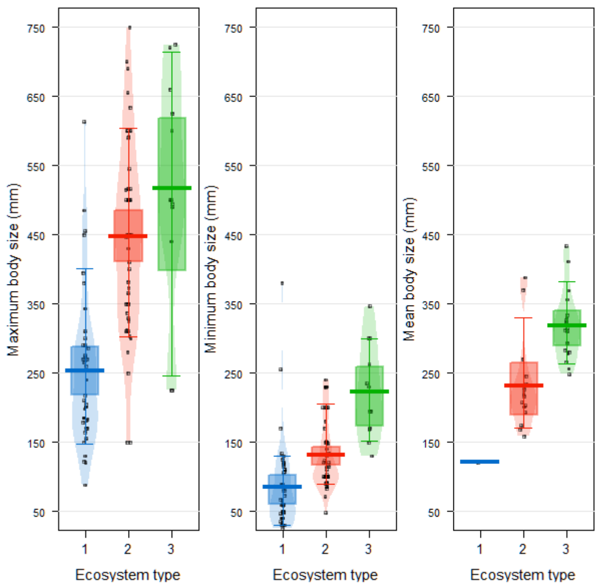

Figure S5B. Brown trout body size (mm) along latitude. Not statistically significant trends were found using generalised additive models (GAMs).

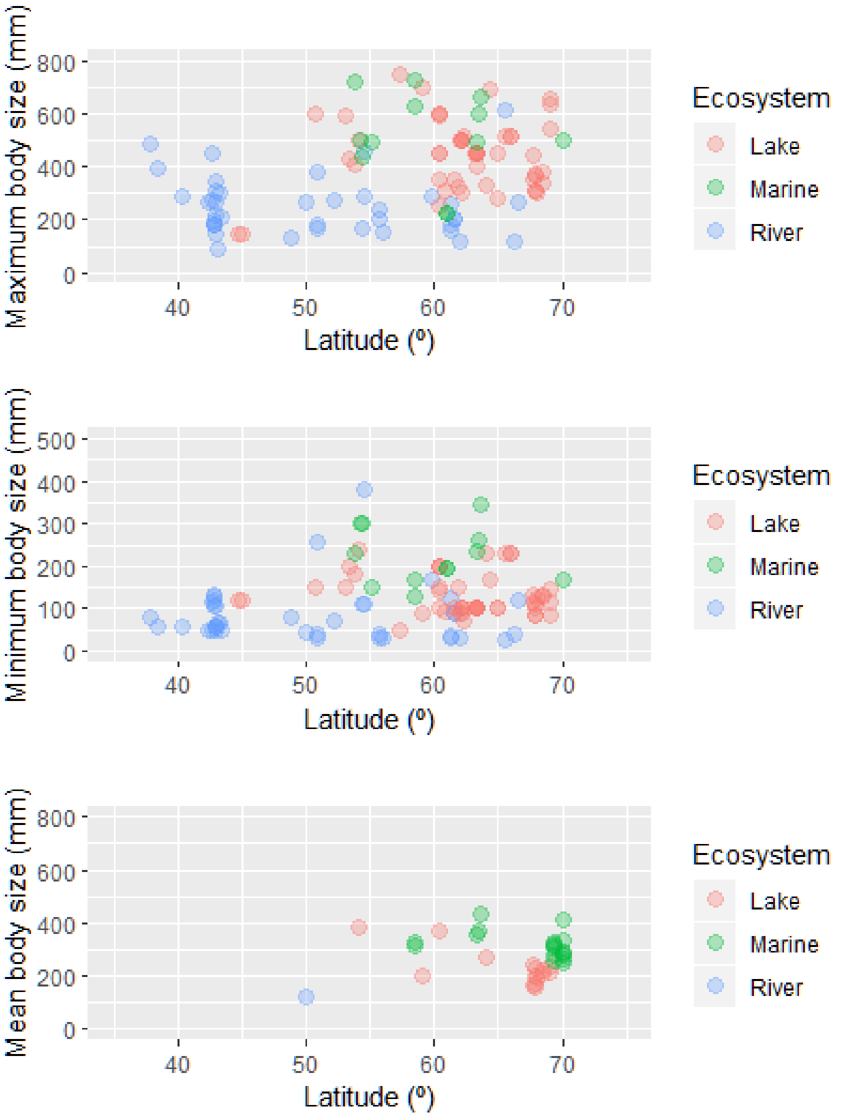

-Appendix S6: Data availability: dataset generated and analysed during the current study. Not specified in the data source (—). Not applicable (NA).

Study	Country	Latitude	Elevation (m)	Ecosystem type	Fish community type	Fish prey type	Piscivory (frequency of occurrence, %)	Fish prey	Fish prey size (mm)	Brown trout size (mm)		
										Minimum	Maximum	Mean
1	Italy	42.55	206	River	Multi-species system	Fish prey and competitors	0	None	NA	—	—	—
2	Norway	61.48	1070	River	One-species system	Cannibalism	0	None	NA	—	—	—
3	Norway	61.95	712	River	Two-species system	Fish prey	0.24	—	NA	30	120	—
4	Norway	61.20	340	River	Two-species system	Fish prey	0	*Phoxinus phoxinus*	—	33	185	—
4	Norway	61.20	340	River	Three-species system	Fish prey	2.5	Minnow	—	35	164	—
5	Czech	50.85	385	River	Two-species system	Fish prey	1.72	*Salvelinus fontinalis*	—	40	170	—
5	Czech	50.87	365	River	Two-species system	Fish competitor	0	None	NA	30	182	—
6	Turkey	38.43	1258	River	Multi-species system	Fish prey	1.31	*Capoeta capoeta*, *Blennius* spp, *Salmo trutta*, *Phoxinellus* spp.	—	57.5	395	—
7	Spain	42.62	854	River	Multi-species system	Fish prey	13.7	—	—	50	450	—
8	Spain	42.34	1140	River	One-species system	Cannibalism	0	None	NA	50	265	—
9	Spain	42.96	430	River	Three-species system	Fish prey	3.6	—	—	60	310	—
10	Spain	42.96	430	River	Three-species system	Fish prey	0.55	*Phoxinus phoxinus, other Cyprinidae*	—	60	150	—
11	UK	50.91	0	River	Multi-species system	Fish prey	0	None	NA	255	380	—
11	UK	54.55	77	River	Multi-species system	Fish prey	5.8	—	—	380	455	—
12	Turkey	37.75	730	River	Multi-species system	Fish prey	3.6	*Blennius* sp., *Salmo trutta*, *Phoxinellus* sp.	—	80	485	—
13	Spain	42.62	940	River	Three-species system	Fish prey and competitors	0	None	NA	115	275	—
14	Spain	42.77	75	River	Two-species system	Fish prey	2.94	*Pseudochondrostoma duriense*	—	119	183	—
14	Spain	42.78	70	River	Two-species system	Fish prey	0	None	NA	133	198	—
14	Spain	42.79	25	River	One-species system	Cannibalism	0	None	NA	106	179	—
14	Spain	42.85	143	River	One-species system	Cannibalism	0	None	NA	130	185	—
14	Spain	43.23	10	River	Two-species system	Fish prey	2.86	*Pseudochondrostoma duriense*	—	65	300	—
14	Spain	42.87	411	River	Two-species system	Fish prey	7.58	*Pseudochondrostoma duriense*	—	48	343	—
14	Spain	42.98	164	River	Two-species system	Fish prey	13.16	*Pseudochondrostoma duriense*	—	59	268	—
14	Spain	42.99	302	River	Two-species system	Fish prey	5.88	*Pseudochondrostoma duriense*	—	108	218	—
15	Spain	40.32	1051	River	Multi-species system	Fish prey	2.4	*Pseudochondrostoma duriense*	—	59	286	—
16	Spain	43.15	395	River	Multi-species system	Fish prey	0.33	*Pseudochondrostoma duriense*	—	67	88	—
17	Iceland	65.58	276	River	Three-species system	Fish prey and competitors	1.2	—	—	25	613	—
18	UK	52.21	156	River	Three-species system	Fish competitor	4.6	*Phoxinus phoxinus*	—	73	274	—
19	Norway	59.88	64	River	Three-species system	Fish competitor	21	*Salmo trutta, Salmo salar*	30–100	170	290	—
20	Czech	49.96	246	River	Multi-species system	Fish prey	0.61	—	—	46	270	120.8
21	Finland	66.28	350	River	Multi-species system	Fish prey	0	None	NA	40	122	—
22	Ukraine	48.73	175	River	Three-species system	Fish prey and competitors	0	None	NA	80	130	—
23	UK	56.06	35	River	Multi-species system	Fish prey and competitors	0	None	NA	30	155	—
24	Sweden	55.68	8	River	One-species system	Cannibalism	0	None	NA	30	205	—
24	Sweden	55.68	8	River	Three-species system	Fish prey	0	None	NA	40	240	—
25	Spain	43.35	93	River	Two-species system	Fish competitor	2.88	—	—	50	210	—
26	Russia	66.55	166	River	Three-species system	Fish prey and competitors	7.35	*Phoxinus phoxinus*	—	120	270	—
27	Norway	61.25	437	River	Multi-species system	Fish prey	0	None	NA	125	260	—
28	UK	54.32	91	River	Three-species system	Fish prey and competitors	2.5	*Salmo trutta, Cottus gobio*	—	110	170	—
28	UK	54.57	210	River	Two-species system	Fish prey	1.1	*Salmo trutta, Cottus gobio*	—	110	290	—
29	Russia	61.57	33	River	Multi-species system	Fish prey	5	—	—	89	201	—
29	Russia	61.57	33	River	Multi-species system	Fish prey	13.64	—	—	89	201	—

30	Norway	67.93	149	Lake	One-species system	Cannibalism	0	None	NA	85	365	173.7
30	Norway	67.90	258	Lake	One-species system	Cannibalism	0	None	NA	110	373	226.5
30	Norway	67.77	72	Lake	Three-species system	Fish prey and competitors	31.3	*Gasterosteus aculeatus*	25–72	134	447	245.4
30	Norway	67.93	284	Lake	Two-species system	Fish competitor	3.2	*Salvelinus alpinus*	87	85	310	158.7
30	Norway	67.83	117	Lake	Three-species system	Fish prey and competitors	14	—	—	103	310	202.9
30	Norway	68.07	35	Lake	Three-species system	Fish prey and competitors	36.2	*Gasterosteus aculeatus*	7–39	123	303	192.6
31	Norway	69.08	125	Lake	Two-species system	Fish competitor	10.8	—	—	114	545.0	233.4
31	Norway	69.11	215	Lake	Three-species system	Fish prey and competitors	6.6	—	—	83	634	216.7
31	Norway	68.50	299	Lake	Two-species system	Fish competitor	4.2	—	—	132	382	220.1
31	Norway	68.51	269	Lake	Two-species system	Fish competitor	5.7	—	—	130	337	206.9
31	Norway	67.78	531	Lake	One-species system	Cannibalism	0	None	NA	115	350	168.4
32	Iceland	64.10	75	Lake	Three-species system	Fish prey and competitors	61	*Gasterosteus aculeatus*, Salmonidae	—	229	329	270
33	UK	54.10	377	Lake	Two-species system	Fish competitor	7.01	*Perca fluviatilis*, *Cottus gobio*	—	240	500	388
34	France	44.72	2490	Lake	Three-species system	Fish prey and competitors	0	None	NA	120	150	—
34	France	44.92	2397	Lake	Three-species system	Fish prey and competitors	0	None	NA	120	150	—
35	UK	57.33	16	Lake	Multi-species system	Fish prey and competitors	33.33	*Salvelinus alpinus*, *Salmo trutta*	—	48	750	—
36	Norway	62.25	856	Lake	Two-species system	Fish competitor	0.33	*Thymallus thymallus*, *Salmo trutta*		71	515	—
37	Finland	64.33	124	Lake	Three-species system	Fish prey	77.26	*Coregonus albula*, *Osmerus eperlanus*	40–120	170	690	—
38	Norway	59.08	281.1	Lake	Three-species system	Fish prey and competitors	68.97	*Coregonus lavaretus*, *Salvelinus alpinus*, *Salmo trutta*	—	90	700	201
39	Norway	62.15	662	Lake	Multi-species system	Fish prey, competitors and predators	59.26	—	—	90	500	—
40	Finland	69.00	148	Lake	Multi-species system	Fish prey, competitors and predators	78.05	*Coregonus lavaretus*, *Pungitius pungitius*, *Salvelinus alpinus*, *Perca fluviatilis*	29–200	148	655	—
41	Norway	61.50	207	Lake	One-species system	Cannibalism	0.4	*Gasterosteus aculeatus*, *Phoxinus phoxinus*, *Salvelinus alpinus*	40–260	100	350	—
41	Norway	60.32	266	Lake	One-species system	Cannibalism	0	None	NA	100	250	—
41	Norway	60.32	734	Lake	Three-species system	Fish prey and competitors	6.6	*Gasterosteus aculeatus*, *Phoxinus phoxinus*, *Salvelinus alpinus*	40–260	140	450	—
41	Norway	63.28	153	Lake	Three-species system	Fish prey and competitors	4.5	*Gasterosteus aculeatus*, *Phoxinus phoxinus*, *Salvelinus alpinus*	40–260	100	450	—
41	Norway	63.32	262	Lake	Two-species system	Fish competitor	0.5	*Gasterosteus aculeatus*, *Phoxinus phoxinus*, *Salvelinus alpinus*	40–260	100	450	—
41	Norway	63.32	298	Lake	Two-species system	Fish competitor	1.7	*Gasterosteus aculeatus*, *Phoxinus phoxinus*, *Salvelinus alpinus*	40–260	100	400	—
41	Norway	63.23	238	Lake	Three-species system	Fish prey and competitors	3.7	*Gasterosteus aculeatus*, *Phoxinus phoxinus*, *Salvelinus alpinus*	40–260	100	450	—
41	Norway	63.27	184	Lake	Three-species system	Fish prey and competitors	0.8	*Gasterosteus aculeatus*, *Phoxinus phoxinus*, *Salvelinus alpinus*	40–260	100	450	—
41	Norway	63.28	168	Lake	Three-species system	Fish prey and competitors	30.1	*Gasterosteus aculeatus*, *Phoxinus phoxinus*, *Salvelinus alpinus*	40–260	100	450	—
41	Norway	64.88	24	Lake	Three-species system	Fish prey and competitors	3.3	*Gasterosteus aculeatus*, *Phoxinus phoxinus*, *Salvelinus alpinus*	40–260	100	450	—
41	Norway	64.90	32	Lake	Three-species system	Fish prey and competitors	0	None	NA	100	280	—
42	Norway	60.33	735	Lake	Three-species system	Fish prey and competitors	14.5	*Phoxinus phoxinus*, *Salvelinus alpinus*	41–199	187	680	370
43	Norway	60.33	735	Lake	One-species system	Cannibalism	0.3	*Phoxinus phoxinus*, *Salvelinus alpinus*	—	—	350	—
43	Norway	60.33	735	Lake	Three-species system	Fish prey and competitors	60	*Phoxinus phoxinus*, *Salvelinus alpinus*	—	200	590	—
43	Norway	60.33	735	Lake	Three-species system	Fish prey and competitors	30	*Phoxinus phoxinus*, *Salvelinus alpinus*	—	200	600	—
43	Norway	60.33	735	Lake	Three-species system	Fish prey and competitors	15	*Phoxinus phoxinus*, *Salvelinus alpinus*	—	150	450	—
44	Faroese-islands	62.13	63	Lake	Two-species system	Fish competitor	0	None	NA	100	500	—
44	Faroese-islands	62.13	76	Lake	One-species system	Cannibalism	0	None	NA	100	300	—
44	Faroese-islands	62.17	25	Lake	One-species system	Cannibalism	0	None	NA	100	500	—
45	Finland	65.57	241	Lake	Multi-species system	Fish prey and competitors	23	*Coregonus alba*, *Pungitius pungitius*, *Perca fluviatilis*	—	230	516	—
45	Finland	66.00	294	Lake	Multi-species system	Fish prey and competitors	91	*Coregonus alba*, *Pungitius pungitius*, *Perca fluviatilis*	—	230	516	—
45	Finland	66.00	294	Lake	Multi-species system	Fish prey and competitors	52	*Coregonus alba*, *Pungitius pungitius*, *Perca fluviatilis*	—	230	516	—
46	Norway	62.00	662	Lake	Multi-species system	Fish prey, competitors and predators	60	*Coregonus lavaretus*, *Salvelinus alpinus*, *Lota tota*, *Salmo trutta*, *Esox lucius*, *Perca fluviatilis*, *Thymallus thymallus*	46–180	90	500	—
47	Germany	50.73	439	Lake	Multi-species system	Fish prey, competitors and predators	35	—	—	150	600	—
48	Norway	61.85	701	Lake	Two-species system	Fish competitor	4.5	—	—	150	325	—
49	Norway	60.80	330	Lake	One-species system	Cannibalism	0	None	NA	92	312	—
50	Finland	64.33	124	Lake	Multi-species system	Fish prey, competitors and predators	90.98	*Coregonus alba*, *Osmerus eperlanus*, *Perca fluviatilis*, *Gyninocephalus cernuss*, *Rutilus rutilus*, *Coregonus lavaretus*,	13–250	—	—	—

								Stizostedion lucioperca, *Pungirius pungitius*				
51	UK	53.35	36	Lake	Two-species system	Fish prey	16.54	*Gasterosteus aculeatus*, *Pungitius gungitius*	—	200	430	—
52	UK	53.10	290	Lake	Two-species system	Fish prey	22.75	*Gasterosteus aculeatus*, *Salmo salar*	—	—	—	—
53	Ireland	53.13	182	Lake	Two-species system	Fish competitor	0.68	*Phoxinus phoxinus*	—	151	592	—
54	Ireland	53.81	60	Lake	Multi-species system	Fish prey	27.1	*Perca fluviatilis*	—	—	—	—
55	Ireland	53.81	60	Lake	Multi-species system	Fish prey, competitors and predators	15.94	*Pungitius gungitius*, *Perca fluviatilis*	—	180	410	—
56	Norway	63.66	0	Marine	Multi-species system	Fish prey, competitors and predators	75	*Sprattus sprattus*, *Clupea harengus*, *Ammodytes tobianus*	—	346	660	433
56	Norway	63.36	0	Marine	Multi-species system	Fish prey, competitors and predators	79.3	*Sprattus sprattus*, *Clupea harengus*, *Ammodytes tobianus*	—	235	495	356
56	Norway	63.48	0	Marine	Multi-species system	Fish prey, competitors and predators	100	*Sprattus sprattus*, *Clupea harengus*, *Ammodytes tobianus*	—	262	600	369
57	UK	54.24	0	Marine	Multi-species system	Fish prey, competitors and predators	45.83	*Clupea* spp., *Pleuronectes flesus*	—	300	500	—
57	UK	54.32	0	Marine	Multi-species system	Fish prey, competitors and predators	47.37	*Clupea* spp., *Pleuronectes flesus*	—	300	440	—
58	Ireland	53.78	0	Marine	Multi-species system	Fish prey, competitors and predators	62.3	*Ammodytes marinus*, *Hyperoplus lanceolatus*, *Sprattus sprattus*, *Clupea harengus*	30–110	230	720	—
59	Ireland	55.16	0	Marine	Multi-species system	Fish prey, competitors and predators	19.49	*Ammodytes* spp., *Atherina presbyter*, *Gadoid* sp., *Percomorph* sp., *Pollachius virens*, *Sprattus sprattus*, *Anguilla anguilla*, *Clupea harengus*, *Gasterosteus aculeatus*, *Pleuronectes platessa*, *Pomatoschistus minutus*, *Trisopterus luscus*	—	150	490	—
60	Norway	58.48	0	Marine	Multi-species system	Fish prey, competitors and predators	23.9	*Sprattus sprattus*, *Clupea harengus*, *Ammodytes* spp., *Coryphopterus flavescens*, *Pomatoschistus minutus*, *Aphia minuta*, *Spinachia spinachia*	—	130	725	314.4
61	Norway	58.48	0	Marine	Multi-species system	Fish prey, competitors and predators	52.8	Clupeiformes, Gadiformes, Gobiidae, Carangdiae, Zoarcidae, Gasterosteidae, Anguillidae, Salmoniformes	—	168	625	326
62	Norway	60.91	0	Marine	Multi-species system	Fish prey, competitors and predators	5.3	—	—	195	225	—
62	Norway	60.91	0	Marine	Multi-species system	Fish prey, competitors and predators	3	—	—	195	225	—
63	UK	56.48	0	Marine	Multi-species system	Fish prey, competitors and predators	30.6	*Clupea harengus*, *Ammodytes* spp.,	—	—	—	—
64	Norway	70.08	0	Marine	Multi-species system	Fish prey, competitors and predators	100	*Clupea harengus*, Gadidae	—	170	500	278
65	Norway	69.37	0	Marine	Multi-species system	Fish prey, competitors and predators	10	*Clupea harengus*	—	—	—	330
65	Norway	69.37	0	Marine	Multi-species system	Fish prey, competitors and predators	90	*Clupea harengus*	—	—	—	310
65	Norway	69.37	0	Marine	Multi-species system	Fish prey, competitors and predators	60	*Clupea harengus*	—	—	—	322
65	Norway	69.37	0	Marine	Multi-species system	Fish prey, competitors and predators	54	*Clupea harengus*	—	—	—	256
65	Norway	69.37	0	Marine	Multi-species system	Fish prey, competitors and predators	89	*Clupea harengus*, *Ammodytes* spp.	—	—	—	280
65	Norway	69.37	0	Marine	Multi-species system	Fish prey, competitors and predators	21	*Clupea harengus*, *Ammodytes* spp., *Pollachius virens*	—	—	—	312
66	Norway	70.08	0	Marine	Multi-species system	Fish prey, competitors and predators	68	*Ammodytes* spp.	—	—	—	334
66	Norway	70.08	0	Marine	Multi-species system	Fish prey, competitors and predators	53	*Ammodytes* spp., *Clupea harengus*	—	—	—	248
66	Norway	70.08	0	Marine	Multi-species system	Fish prey, competitors and predators	65	*Ammodytes* spp., *Clupea harengus*	—	—	—	266
66	Norway	70.08	0	Marine	Multi-species system	Fish prey, competitors and predators	81	*Ammodytes* spp., *Clupea harengus*, Gadidae, Capelin	—	—	—	283
66	Norway	70.08	0	Marine	Multi-species system	Fish prey, competitors and predators	86	*Ammodytes* spp., *Clupea harengus*, Gadidae	—	—	—	411
66	Norway	70.08	0	Marine	Multi-species system	Fish prey, competitors and predators	72	*Ammodytes* spp., *Clupea harengus*, Gadidae	—	—	—	293

1. Fochetti, R., Argano, R. & Tierno de Figueroa, J.M. (2008). Feeding ecology of various age-classes of brown trout in River Nera, Central Italy. Belgian Journal of Zoology, 138: 128–131.
2. Hesthagen, T. & Fjellheim, A. (1987). Effects of transferring glacier-fed water to a clear-water mountain river on the production and food organisms of brown trout (*Salmo trutta* L.) in Southern Norway. Regulated Rivers: Research & Management, 1: 161–170.
3. Hesthagen, T., Saksgård, R., Hegge, O., Dervo, B.K. & Skurdal, J. (2004). Niche overlap between young brown trout (*Salmo trutta*) and Siberian sculpin (*Cottus poecilopus*) in a subalpine Norwegian river. Hydrobiologia, 521: 117–125.
4. Holmen, J., Olsen, E.M. & Vøllestad, L.A. (2003). Interspecific competition between stream-dwelling brown trout and Alpine bullhead. Journal of Fish Biology, 62: 1312–1325.
5. Horká, P., Sychrová, O., Horký, P., Slavík, O., Švátora, M., & Petrusek, A. (2017). Feeding habits of the alien brook trout *Salvelinus fontinalis* and the native brown trout *Salmo trutta* in Czech mountain streams. Knowledge & Management of Aquatic Ecosystems, 418, 1–11
6. Kara, C. & Alp, A. (2005). Feeding habits and diet composition of brown trout (*Salmo trutta*) in the upper streams of River Ceyhan and River Euphrates in Turkey. Turkish Journal of Veterinary and Animal Sciences, 29: 417–428.

7. López-Alvarez, J.V. (1984). Observaciones sobre la alimentación natural de la trucha comun (*Salmo trutta fario* L.) en algunos rios de la Cuenca del Duero. Limnetica 1: 247–255.
8. Montori, A., Tierno de Figueroa, J.M. & Santos, X. (2006). The diet of the brown trout *Salmo trutta* (L.) during the reproductive period: size-related and sexual effects. International Review of Hydrobiology, 91: 438–450.
9. Oscoz, J., Escala, M.C. & Campos, F. (2000). La alimentación de la trucha común (*Salmo trutta* L., 1758) en un río de Navarra (N. España). Limnetica, 18: 29–35.
10. Oscoz, J., Leunda, P.M., Escala, M.C. & Miranda, R. (2008). Summer feeding relationships of the co-occurring hatchling brown trout *Salmo trutta* and Ebro minnows *Phoxinus bigerri* in an Iberian river. Acta Zoologica Sinica, 54: 675–685.
11. Pentelow, F.T.K. (1932). The Food of the Brown Trout (*Salmo trutta* L.). Journal of Animal Ecology, 1: 101–107.
12. Alp, A., Kara, C. & Buyukçapar, H. M. (2005). Age, growth and diet composition of the resident brown trout *Salmo trutta macrostigma* Dumeril 1858, in Firniz stream of the river Ceyhan, Turkey. Turkish Journal of Veterinary and Animal Sciences, 29: 285–295.
13. Rocaspana, R., Aparicio, E., Vinyoles, D. & Palau, A. (2016). Effects of pulsed discharges from a hydropower station on summer diel feeding activity and diet of brown trout (*Salmo trutta* Linnaeus, 1758) in an Iberian stream. Journal of Applied Ichthyology, 32: 190–197.
14. Sánchez-Hernández, J. (2009). Biología de la alimentación de la trucha común (*Salmo trutta* Linné, 1758) en los ríos de Galicia. Thesis. Universidad de Santiago de Compostela, ISBN: 978-84-9887-188-3.
15. Sánchez-Hernández, J & Cobo, F. (2011). Summer food resource partitioning between four sympatric fish species in Central Spain (River Tormes). Folia Zoologica, 60: 189–202.
16. Sánchez-Hernández, J., Vieira-Lanero, R., Servia, M.J. & Cobo, F. (2011). Feeding habits of four sympatric fish species in the Iberian Peninsula: keys to understanding coexistence using prey traits. Hydrobiologia, 667:119–132.
17. Steingrímsson, S.Ó. & Gíslason, G.M. (2002). Body size, diet and growth of landlocked brown trout, *Salmo trutta*, in the subarctic River Laxá, North-East Iceland. Environmental Biology of Fishes, 63: 417–426.
18. Thomas, J.D. (1962). The food and growth of brown trout (*Salmo trutta* L.) and its feeding relationships with the salmon parr (*Salmo salar* L.) and the eel (*Anguilla anguilla* (L.)) in the River Teifi, west Wales. Journal of Animal Ecology. 31, 175-205.
19. Vik, J.O., Borgstrøm, R. & Skaala, Ø. (2001). Cannibalism governing mortality of juvenile brown trout, *Salmo trutta*, in a regulated stream. Regulated Rivers: Research & Management, 17: 583–594.
20. Vlach, P., Švátora, M. & Dušek, J. (2013). The food niche overlap of five fish species in the Úpoř brook (Central Bohemia). Knowledge and Management of Aquatic Ecosystems, 411, 04.
21. Kreivi, P., Muotka, T., Huusko, A., Mäki-Petäys, A., Huhta, A. & Meissner, K. (1999). Diel feeding periodicity, daily ration and prey selectivity in juvenile brown trout in a subarctic river. Journal of Fish Biology, 55: 553–571.
22. Kruzhylina, S. & Didenko A. (2011). Autumn diet and trophic relations of juvenile Brown trout (*Salmo trutta*), Rainbow trout (*Oncorhynchus mykiss*) and European grayling (*Thymallus thymallus*) in the Shipot River (Ukraine), Transylvanian Review of Systematical and Ecological Research, 11: 169–182.
23. Maitland, P. S. (1965). The feeding relationships of salmon, trout, minnows, stone loach and three-spined sticklebacks in the River Endrick, Scotland. Journal of Animal Ecology, 34: 109–133.
24. Otto, C. (1976). Size, growth, population density and food of brown trout *Salmo trutta* L. in two sections of a south Swedish stream. Journal of Fish Biology, 8: 477–488.
25. Suárez, J.L., Reiriz, L. & Anadón, R. (1988). Feeding relationships between two salmonid species and the benthic community. Polish Archives of Hydrobiology, 35: 341–359.
26. Yevsin V.N. & Ivanov N.O. (1979). The summer feeding of brown trout, *Salmo trutta*, in the Pulon'ga river (Kola Peninsula). Journal of Ichthyology, 19, 122–127.
27. Jonsson, B. & Sandlund, O.T. (1979). Environmental factors and life histories of isolated river stocks of brown trout (*Salmo trutta m. fario*) in Søre Osa river system. Norway Environmental Biology of Fishes, 4: 43–54.
28. McCormack, J. C. (1962). The food of young trout (*Salmo trutta*) in two different becks. Journal of Animal Ecology, 31: 305–316.
29. Shustov, Yu. A., Veselov, A.E. & Baryshev, I.A. (2008). The diet of juvenile lake trout *Salmo trutta* L. in rivers of the Onega basin in autumn. Russian Journal of Ecology, 39: 119–122.
30. Sánchez-Hernández, J. & Amundsen, P.-A. (2015). Trophic ecology of brown trout (*Salmo trutta* L.) in subarctic lakes. Ecology of Freshwater Fish, 24: 148–161.
31. Sánchez-Hernández, J., Eloranta, A.P., Finstad, A.G. & Amundsen, P.-A. (2017). Community structure affects trophic ontogeny in a predatory fish. Ecology and Evolution, 7: 358–367.
32. Björnsson, B. (2001). The trophic ecology of Arctic charr (*Salvelinus alpinus*) and Brown trout (*Salmo trutta*) in Ellidavatn, a small lake in Southwest Iceland. Limnologica, 31: 199–207.
33. Burrough, R. J. & Kennedy, C. R. (1978). Observations on the brown trout (*Salmo trutta*) and perch (*Perca fluviatilis*) of Malham Tarn, North Yorkshire. Field Studies, 4: 631–643.

34. Cavalli, L., Chappaz, R. & Gilles, A. (1998). Diet of Arctic charr (*Salvelinus alpinus* (L.)) and brown trout (*Salmo trutta* L.) in sympatry in two high altitude alpine lakes. Hydrobiologia, 386: 9–17.
35. Grey, J. (2001). Ontogeny and dietary specialization in brown trout (*Salmo trutta* L.) from Loch Ness, Scotland, examined using stable isotopes of carbon and nitrogen. Ecology of Freshwater Fish, 10: 168–176.
36. Haugen, T.O. & Rygg, T.A. (1996). Food- and habitat-segregation in sympatric grayling and brown trout. Journal of Fish Biology, 49: 301–318.
37. Hyvärinen, P. & Huusko, A. (2006). Diet of brown trout in relation to variation in abundance and size of pelagic fish prey. Journal of Fish Biology, 68: 87–98.
38. Jensen, H., Kiljunen, M. & Amundsen, P.-A. (2012). Dietary ontogeny and niche shift to piscivory in lacustrine brown trout *Salmo trutta* revealed by stomach content and stable isotope analyses. Journal of Fish Biology, 80: 2448–2462.
39. Jonsson, N., Næsje, T.F., Jonsson, B., Saksgård, R. & Sandlund, O.T. (1999). The influence of piscivory on life history traits of brown trout. Journal of Fish Biology, 55: 1129–1141.
40. Kahilainen, K. & Lehtonen, H. (2003). Piscivory and prey selection of four predator species in a whitefish dominated subarctic lake. Journal of Fish Biology, 63: 659–672.
41. L'Abée-Lund, J.H., Langeland, A. & Sægrov, H. (1992). Piscivory by brown trout *Salmo trutta* L. and Arctic charr *Salvelinus alpinus* (L.) in Norwegian lakes. Journal of Fish Biology, 41: 91–101.
42. L'Abée-Lund, J.H., Aass, P. & Sægrov, H. (1996). Prey orientation in piscivorous brown trout. Journal of Fish Biology, 48: 871–877.
43. L'Abée-Lund, J.H., Aass, P. & Sægrov, H. (2002). Long-term variation in piscivory in a brown trout population: effect of changes in available prey organisms. Ecology of Freshwater Fish, 11: 260–269.
44. Malmquist, H., Ingimarsson, F., Jóhansdóttir, E.E., Gíslason, D. & Snorrason, S.S. (2002). Biology of brown trout (*Salmo trutta*) and Arctic charr (*Salvelinus alpinus*) in four Faroese lakes. Annales Societatis Scientiarum Færoensis, 36: 94–113.
45. Niva, T. (1999). Relations between diet, growth, visceral lipid content and yield of the stocked brown trout in three small lakes in northern Finland. Annales Zoologici Fennici, 36: 103–120.
46. Næsje, T.F., Sandlund, O.T. & Saksgaard, R. (1998). Selective predation of piscivorous brown trout (*Salmo trutta* L.) on polymorphic whitefish (*Coregonus lavaretus* L.). Archiv für Hydrobiologie Special Issues in Advanced Limnology 50: 283–294.
47. Radke, R.J., Kahl, U. & Benndorf, J. (2003). Food-web manipulation of drinking water reservoirs with salmonids: vertical distribution of prey and predator. Limnologica, 33: 92–98.
48. Saksgård, R. & Hesthagen, T. (2004). A 14-year study of habitat use and diet of brown trout (*Salmo trutta*) and Arctic charr (*Salvelinus alpinus*) in Lake Atnsjøen, a subalpine Norwegian lake. Hydrobiologia, 521, 187–199.
49. Schei, T.A. & Jonsson, B. (1989). Habitat use of lake-feeding, allopatric brown trout in Lake Oppheimsvatnet, Norway. Pp. 156-168. IN: Brannon, E., Jonsson, B. (Eds.). Proceedings of the salmonid migration and distribution symposium. University of Washington, Seattle.
50. Vehanen, T., Hyvärinen, P. & Huusko, A. (1998). Food consumption and prey orientation of piscivorous brown trout (*Salmo trutta*) and pikeperch (*Stizostedion lucioperca*) in a large regulated lake. Journal of Applied Ichthyology, 14: 15–22.
51. Hunt, P.C. & Jones, J.W. (1972). The food of brown trout in Llyn Alaw, Anglesey, North Wales. Journal of Fish Biology, 4: 333–352.
52. Pedley, R.B. & Jones, J.W. (1978). The comparative feeding behaviour of brown trout, *Salmo trutta* L. and Atlantic salmon, *Salmo salar* L. in Llyn Dwythwch, Wales. Journal of Fish Biology, 12: 239–256.
53. Moriarty, C. (1963/1964). Food of Perch (*Perca fluviatilis*, L.) and Trout (*Salmo trutta*, L.) in an Irish Reservoir. Proceedings of the Royal Irish Academy. Section B: Biological, Geological, and Chemical Science. 63: 1-31.
54. Gargan P.G. & O'Grady M.F. (1992). Feeding relationships of trout *Salmo trutta* L., perch *Perca fluviatilis* (L.) and roach *Rutilus rutilus* (L.) in Lough Sheelin, Ireland. Irish Fisheries Investigations, 35A: 1–22.
55. Fitzmaurice, P. (1979). Selective predation on Cladocera by brown trout *Salmo trutta* L. Journal of Fish Biology 15: 521–525.
56. Davidsen, J.G, Knudsen, R., Power, M., Sjursen, A.D., Rønning, L., Hårsaker, K., Næsje, T. & Arnekleiv, J.V. (2017). Trophic niche similarity among sea trout *Salmo trutta* in Central Norway investigated using different time-integrated trophic tracers. Aquatic Biology, 26: 217–227.
57. Elliott, J.M. (1997). Stomach contents of adult sea trout caught in six English rivers. Journal of Fish Biology, 50: 1129–1132.
58. Fahy, E. (1983). Food and gut parasite burden of migratory trout *Salmo trutta* L. in the sea. Irish Naturalists' Journal 21, 11–18.
59. Fahy, E. (1985). Feeding, growth and parasites of trout *Salmo trutta* L. from Mulroy Bay, an Irish sea lough. Irish Fisheries Investigations, 25: 1–12.
60. Knutsen, J.A., Knutsen, H., Gjøseter, J. & Jonsson, B. (2001). Food of anadromous brown trout at sea. Journal of Fish Biology 59, 533–543

61. Knutsen, J.A., Knutsen, H., Olsen, E.M. & Jonsson, B. (2004). Marine feeding of anadromous *Salmo trutta* during winter. Journal of Fish Biology, 64: 89–99.
62. Lyse, A.A., Stefansson, S.O. & Fernö, A. (1998). Behaviour and diet of sea trout post-smolts in a Norwegian fjord system. Journal of Fish Biology, 52: 923–936.
63. Pemberton, R. (1976). Sea trout in North Argyll sea lochs. II. diet. Journal of Fish Biology, 9: 195–208.
64. Rikardsen, A.H. & Amundsen, P.-A. (2005). Pelagic marine feeding of Arctic charr and sea trout. Journal of Fish Biology, 66: 1163–1166.
65. Rikardsen, A.H., Amundsen, P.-A., Knudsen, R. & Sandring, S. (2006). Seasonal marine feeding and body condition of sea trout *Salmo trutta* (L.) at its northern distribution area. ICES Journal of Marine Science, 63, 466–475.
66. Rikardsen, A.H., Dempson, J.B., Amundsen, P.-A., Bjørn, P.A., Finstad, B. & Jensen, A.J. (2007). Temporal variability in marine feeding of sympatric Arctic charr and sea trout. Journal of Fish Biology, 70: 837–852.

-Appendix S7: Meta-regression

A 'funnel' plot

The trim-and-fill analysis indicates 43 missing essays on the right (white circles) suggesting a small publication bias on the left (i.e. negative effect sizes). Thus, i.e. there is visual indication of publication bias and between-study heterogeneity (Figure 7A).

Figure 7A. Funnel plot illustrating the relationship between effect size (Log Odds ratio) and standard error, where each black point is an individual study on the relationship between piscivory and ecosystem type.

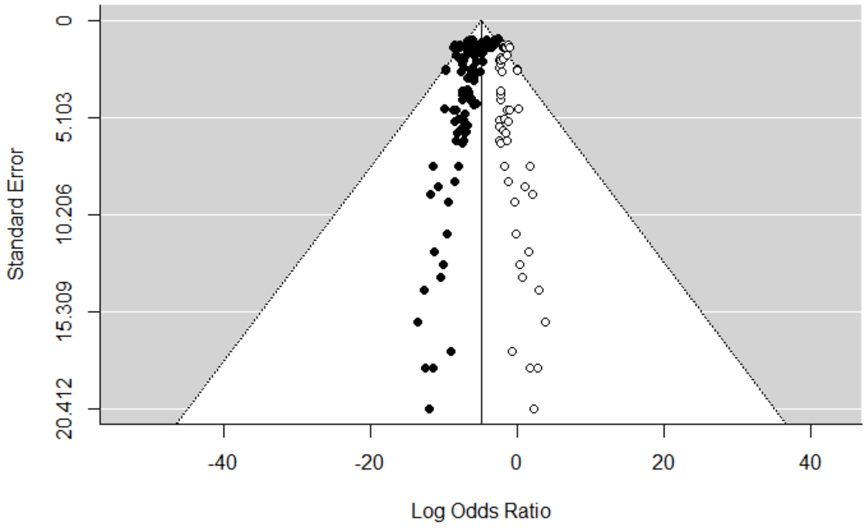

Meta-analysis (mixed-effect models varying moderators)

Meta-analysis of the odds ratios using a random-effects models (meta-regression) revealed that larger magnitudes of effect sizes tend to increase from freshwater to marine systems, whereas no significant trends were found using the other covariates (fish community type, fish prey system, latitude, elevation and body size) as moderators. Indeed, the model with ecosystem type as moderator showed the highest amount of heterogeneity accounted for (55.5%, see summary table below).

Figure 7B. Meta-analysis of the effect size (odds ratios) using a random-effects model using covariates (ecosystem type, fish community type, latitude and body size) as a moderator. Fitted values (red line) with 95% confidence bands (broken black line) are shown only for statistically significant trends. The sizes of the bubbles reflect the inverse of the standard errors (i.e. more precise studies are shown as bigger points).

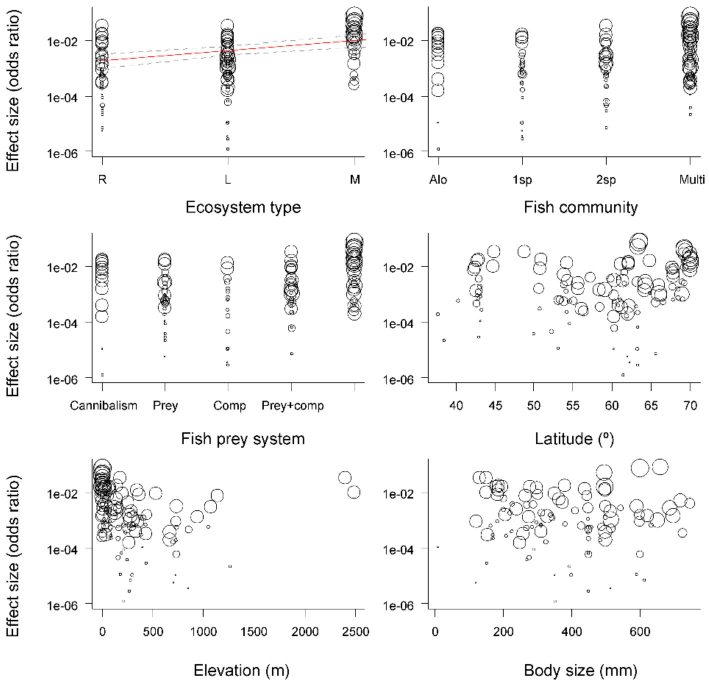

Table 7. summary table of the mixed-effect models varying moderators

tau^2 = estimated amount of residual heterogeneity
tau = square root of estimated tau^2 value
I^2 = residual heterogeneity / unaccounted variability (%)
H^2 = unaccounted variability / sampling variability
R^2 = amount of heterogeneity accounted for (%)

	Model results	Tests		Amount of heterogeneity				
	Estimate	Residual heterogeneity	Moderator	tau^2 (± SE)	tau	I^2	H^2	R^2
**Model 1**								
Intercept	-7.104***	*P* = 0.902	***P* < 0.001**	0.3799 ± 0.3942	0.6164	11.20%	1.13	55.55%
Moderator (ecosystem type)	0.814***							
**Model 2**								
Intercept	-5.793***	*P* = 0.569	*P* = 0.523	0.8086 ± 0.4429	0.8992	21.19%	1.27	3.33%
Moderator (fish community type)	0.109							
**Model 3**								
Intercept	-6.243***	*P* = 0.685	*P* = 0.070	0.696 ± 0.4225	0.8342	18.78%	1.23	16.80%
Moderator (fish prey system)	0.223							
**Model 4**								
Intercept	-7.059***	*P* = 0.604	*P* = 0.227	0.7587 ± 0.4387	0.8864	20.71%	1.26	6.06%
Moderator (latitude)	0.027							
**Model 5**								
Intercept	-5.301***	*P* = 0.627	*P* = 0.169	0.7601 ± 0.4341	0.8718	20.18%	1.25	9.12%
Moderator (elevation)	-0.001							
**Model 6**								
Intercept	-5.524***	*P* = 0.804	*P* = 0.693	0.8272 ± 0.5254	0.9095	19.26%	1.24	0.00%
Moderator (body size)	-0.001							

-Appendix S8: Multicollinearity (variance inflation factors -VIF)

Multicollinearity between latitude and the other quantitative variables (elevation, annual mean temperature, temperature annual range, temperature seasonality, temperature of warmest month and mean temperature of warmest quarter). *Multicollinearity in marine ecosystems was tested without elevation as all data records included sampling at sea level.

	Riverine	Lacustrine	Marine*	Total (pooled data)
Body size (maximum length)	1.550597	1.544507	3.80252	1.205086
Elevation (m)	2.733977	4.455040	—	1.905576
Annual mean temperature (BIO1)	671.806579	517.797928	2568.30968	617.062618
Temperature annual range (BIO2)	29.437763	29.180870	187.94199	617.062618
Temperature seasonality (BIO4)	173.843316	318.251932	1417.03506	24.864381
Max temperature of warmest month (BIO5)	220.964323	314.293245	2909.38424	275.577759
Mean temperature of warmest quarter (BIO10)	670.410144	504.395020	2494.73050	689.058763
